# Spirit of the Foreign Periodicals, &c.

**Published:** 1842-04-01

**Authors:** 


### Spirit of the Foreign Periodicals.

### Prefatory Remarks.

In our last number we made our readers acquainted with the observations of an intelligent German physician on the general character of French medical practice and literature, and we took that opportunity of pointing out the marked coincidence of his opinions with those which have been expressed by ourselves for many years past in the pages of this Journal. The study of national, as of individual, differences is always an interesting, and often a very instructive one; and certainly in no department of human knowledge is the importance of it more prominently true than in medicine. For not only is there something peculiar in the original constitution of almost every patient, manifested in almost every complaint with which he may be affected, but there is a host of other influences, some internal or appertaining to the person himself, and others external or connected with his mode of life, occupation, season, climate and so forth, each and all of which have a tendency to modify the phenomena of morbid action, and the consideration of which will never be overlooked by the scientific physician. It is vain therefore to seek to establish fixed and unalterable laws in medical as in the exact sciences. Then, again, how multiform and ever-varying are the forms of those ills to which our bodies are liable! Though seldom perfectly alike, there is always some feature of resemblance in their symptoms or in their mode of progress:

——— facies non omnibus una,
Nec diversa tamen.———

Are not some endemic, and obviously dependent upon the agency of a local cause, while others are met with in every corner of the world? Is not the same (apparently) disease at one time of casual occurrence and at another time of wide-spread and epidemic extent? Are not some maladies contagious and others not so? Is not the course of the former usually more regular and defined in their successive stages than the latter? Are there not certain diseases which are developed only once during life, while there is no limit to the recurrence of others? These are only a few out of numerous other questions, connected with the history of our science, which will perhaps ever continue to baffle and perplex the physician: there are, we may be assured, mysteries in medicine, as there are in religion; and the part of the wise man is to acknowledge, although he cannot explain them.

But leaving these generalities, let us consider for a moment how numerous are the influences of a more local and circumscribed nature, which tend to introduce discrepancy and confusion of opinion among medical men in their study of diseases. We have already said that the constitutions, like the faces, of no two persons are exactly and in all respects alike. If this be true of individuals, how much more forcibly true is it of nations. No two people fare entirely alike, or breathe exactly the same atmosphere, or live on the same food, or are clothed with the same raiment, or are exposed to the same vicissitudes of weather. Of late years the study of the differences of temperaments, natural or acquired, has been far too much overlooked; and hence some physicians of the modern hasty school have vainly attempted to apply exactly the same line of treatment to nearly all patients alike affected with a disease, in all seasons and in all climates. The "school-master abroad," that we have of late years heard so much about, might have taught medical men a wiser lesson. Surely the severity of any kind of discipline should be regulated by the character of each individual! The same amount of depletion, or of stimulation, which may be suited to one, may be hurtful to another. Much will depend on the mode of living too of the parties; on their previous health; on their moral temperament; on their occupations, &c. Now these considerations ought unquestionably not to be lost sight of in the study of diseases as they appear in different seasons and in different climates; and yet it must be confessed that in the present day we very rarely meet with any marked allusion to them. For many years past medical literature has been almost exclusively occupied with what have been called practical treatises on this or on that malady; which treatises are too often little more than the records of the experience of some physician or surgeon in a particular disease during a very limited period of time. We do not require to be told that the aim and object of all theory and study is to improve our practice and to render the treatment of diseases more uniformly successful; but we have great doubts whether the authors of the present century have been taking the right way to achieve this much-desired end. How comes it, we ask, that not a year passes but some work comes out questioning or positively contradicting the facts and reasonings of a production of the preceding one? Are we in truth at all more successful in the treatment of some of the most common and fatal diseases than were our predecessors fifty or sixty years ago? Is the treatment of typhus fever, for example, better understood than it was in the days of Cullen and Pitcairn? or rather have we not, after trying a variety of new-fangled experiments of our own, been of late returning to the principles and precepts of these and such-like men? Even in that class of diseases, in a correct knowledge of which more progress has been made during the last 30 years than in any other—we allude to the diseases of the lungs and heart—the stability and success of our modes of treatment have not made corresponding advances with our improved means of diagnosis. We frankly and unhesitatingly admit that the stethoscope has done a vast deal for the improvement of medicine; but we must not blind ourselves against the staring fact that the *use* of it has in a thousand instances led the physician to erroneous conclusions. It was only the other day that a friend of our's consulted a celebrated stethoscopist—and well-known writer on auscultation—of this metropolis for palpitation of the heart; the case was pronounced after due examination to be one of hypertrophe and incipient disease in the valves of this organ. Fortunately, however, for our friend, who is of a highly excitable temperament, the palpitations gradually subsided of themselves, and there is now not the slightest reason to suspect any cardiac disorder. Then again, on the subject of renal disease, has there not been, of late years, a most extravagant disposition in some even of our best physicians to magnify the frequency and importance of an alleged lesion in the structure of the kidneys as the cause of a multitude of diseases? We know of not a few instances in which the case has been pronounced to be one of the *morbus Brightii*, from the mere occasional presence of a portion of albumen in the urine, when its other symptoms, as well as its ultimate issue, have pretty satisfactorily proved that the kidneys were little, if at all, at fault. Mistakes of this sort are of daily occurrence, and are, alas! sometimes attended with serious consequences. We blame not the stethoscope and its truly great inventor, nor the test-tube and such writers as *Bright*, *Christison*, and *Rayer*, for these mistakes; but we certainly attach a great deal of the blame to the prevailing character of medical writings and medical lectures in the present day. They are almost all calculated to exaggerate the importance of one set of symptoms or of one mode of treatment, and are therefore greatly deficient in those philosophic and comprehensive views of disease which pervade the writings of the preceding generation. Well would it be for our science if some one, akin to the master-minds of last century, such as *Boerhaave*, or *Cullen*, or *Hunter*, or *Bichat*, were to arise and review the phases which have taken place during the course of the present one. There seems however, little prospect of such an event; the aim and end of all appearing to be that of brother Jonathan in his commercial pursuits, "to go ahead" of all competitors for present wealth and fame. The task is one, too, which requires a rare union of high intellectual and moral endowments; a thorough practical knowledge of diseases, an extensive and refined scholarship, a penetrating sagacity of view, and a calm impartiality of judgment—not omitting the necessary "otium cum dignitate," and a sincere love of the subject.* How worthy such a labour would be of an *Abercrombie* or an *Alison*—the two men in this country who could probably do it most justice!

It is certainly much to be regretted that the great captains of our art should so rarely communicate the general results and mature after-thoughts, so to speak, of their experience and observation in the form, not of detailed treatises on any particular disease, but of comprehensive commentaries or essays, for the benefit of those who come after them. We strongly suspect, that if any of them were doing so in the present day, their judgment would not be very flattering to the medical works of the present century, and that, while they acknowledged the progress that had been made in some very useful manipulations, they would tell us that there was little of that dignity of design and breadth of execution which stamp the productions of a former age. There has been much neat chiselling and clever masonry in this age; but where are the mighty temples and the god-like statues of antiquity? One reason, among others, of the want of that philosophic comprehensiveness in medical works of the present century, is unquestionably the neglect of the past literature of the profession. In forgetting our forefathers we have unduly magnified ourselves.

We were certainly no less pleased than surprised, the other day, with meeting the letter of M. *Double*, that accomplished physician of the French metropolis, on the study of the ancient classical authors of medical literature, and from which we have given copious extracts in a future page. Many, we know, will be inclined to say, that he surely exaggerates the value of such pursuits; but we should suggest to such persons that it may be wise to hesitate in their judgment, when they find a man, an acknowledged master in the practical duties of his profession, confessing that he often falls back upon the pages of Hippocrates and Galen for instruction as well as for gratification.

---

* It is not by poets alone, while engaged in the preparation of any great work, that the fine remarks of *Milton* should be borne in mind:........ "An inward prompting grew daily upon me that by labour and intent study, which I take to be my portion in this life, joined to the strong propensity of nature, I might, perhaps, leave something so written to after-times, as that they should not willingly let it die."...... "The accomplishment of these intentions lies not but in a power above man's to promise; but that none hath by more studious ways endeavoured, and with more unwearied spirit that none shall, that I dare almost to aver of myself, so far as life and free leisure shall extend. Neither do I think it shame to covenant with any knowing reader, that some few years yet I may go in trust with him toward the payment of that for which I am now indebted; as being a work not to be raised from the heat of youth or the vapours of wine, like that which flows at waste from the pen of some vulgar amorist, nor to be obtained by the invocation of dame memory and her syren daughters; but by devout prayer to that Eternal Spirit, who can enrich with all utterance and knowledge, and sends out his Seraphim with fire from his altar to touch and purify the lips of whom he pleases."

What a magnificent introduction of a work! how worthy is the vestibule of the sublime temple that was reared by

"The blind old man of *Britain's* rocky isle!"

In the discussion, which took place in the Royal Academy on the subject connected with M. *Double's* letter, we find quite such a discrepancy of opinions as we might have anticipated from our knowledge of the members of that learned body. The physical-force physicians, of which M. *Bouillaud* may fairly be considered as one of the leaders, at once asserted that there was nothing in any of the ancient authors that was not a great deal better said in the writings of the present day, that almost all their theories were vague and foolish, and that their practice was, as a matter of course, worthy only of old women.*

It is fortunate, however, that all do not form quite so summary a judgment of the question as the disciples of the so-called physiological school. A very intelligent writer in the Gazette Medicale, has made some excellent remarks on the subject.

"We have heard," says he, "some persons regret that the Academy should waste its time in such controversies. These persons are greatly mistaken. There is no reason to believe that the communications suspended by this literary episode would have offered any greater interest. Such discussions elevate in our opinion not only the Academy, but also medical science, of which the Academy is at once the organ, the focus, and the interpreter. Let us remember, that medicine, viewed in its comprehensive range, is an elevated and complex science, which requires in its true disciples the union of the artist and the philosopher, and the most varied development of the higher faculties of the intellect applied to the examination of all the moral and physical circumstances which from far or near affect mankind. It is thus that medicine is eminently distinguished from all the purely physical and mechanical sciences, and becomes intimately allied with the metaphysical, moral and social sciences, with the general philosophy of man, with the history of the species, and, by a necessary consequence, with literature and erudition. If, therefore, modern medicine wishes not to lag behind its high functions, and hopes to maintain its intellectual supremacy, it must firmly resist that hurtful tendency, so prevalent in the present day, to push science on to a 'material positivism,' the immediate practical value of which, however highly it may be thought of, can never compensate for a more serious injury, that of debasing and confining the faculties of the mind."........

"Even if we were to admit that we could dispense entirely with ancient medicine, we should not break off our acquaintance with the physicians of those days; an intercourse with the great minds, which have successively enriched the field of science, is always profitable. There is always something to be gained from a communion with such men as Hippocrates, Galen, and Boerhaave. The errors of such men do not resemble those which common minds fall into; they almost always contain some profound thought, or suggest some useful reflection."...... ...

"If the medicine of the present century has, in many respects, left far behind that of former times, it is by no means as certain that the physicians of our day can be individually compared with those of past ages. At no period have literary studies and pursuits been more neglected than they are now. If we wanted a proof of this, we should only have to point to the discussion which recently took place in the Academy. Very few of the members ventured to

---

* Another learned (!) academician, M. *Bouvier*—is this the gentleman who a year or two was boasting that he could straiten, by one application of a powerfully-extending mechanical apparatus, joints that had been contracted for years?—assured his audience that the only way to admire Hippocrates was not to read his works; for that, having one day looked into them by chance, he found nothing but absurd peculiarities and the most complete *galimatias* in every page!

take part in it; and of the few that did, there was scarcely more than one or two who seemed to have any knowledge of the writers of antiquity.

Even if they had perused no more than the beautiful introduction to the works of Hippocrates by M. *Littre,* the most recent and the most remarkable work on the Coan sage and his writings, they would have made a more respectable appearance. As to the critical and philosophical opinions expressed by most of the speakers, it was really painful to listen to them; and we know not whether to be more surprised at the incredible penury of their actual knowledge, and the feeble and limited style of their reasoning, or at the confident assurance with which they spouted forth their ignorance. We blame not them alone for this state of things; but we blame the vicious state of modern education, which, being exclusively occupied with the more immediate requirements of our art, calls into play those faculties only necessary for this purpose, and dispenses with all those branches of knowledge which do not directly appertain to it. The interest of the art is thus sacrificed to the interest of the artist; and hence the mass of medical practitioners are, it must be confessed, sadly deficient in some of the most important qualifications of an enlightened and well-educated gentleman. We may rest assured that the social position of medical men, even in reference to mere worldly matters, will be improved just in proportion as they distinguish themselves by their intellectual and moral attainments, and that their profession will be the more honoured and rewarded as their characters are more dignified above the other classes of society."

Several of the articles in the Foreign Periscope of our present number will enable the reader to form a tolerably fair opinion of the characters of some of the leading physicians and surgeons in France of late years, and of the general tone of professional feeling that has existed for the last thirty years and is still dominant in that country.

The first article deserves especial notice from the great interest of the subject —Sir *C. Bell's* celebrated discovery in respect to the different functions of the roots of the spinal nerves. Like its great predecessor in the annals of physiology, the circulation of the blood, this discovery, after encountering all sorts of opposition and contradiction for a length of time, seems to be now admitted even by the French school—the last, indeed, to acknowledge its truth,—to be firmly and irremovably established.

The experimental researches of M. *Longet* have been very numerous, and are perfectly conclusive. In our opinion they were unnecessary; but the French writers think otherwise, and, with a truly national spirit, they appear to intimate that, until the publication of M. *Longet's* work, and the recent communication of M. *Begin* to the Royal Academy, Sir *C. Bell's* doctrine on the Nervous System had not been at all satisfactorily proved. It is unnecessary here to dispute their claims; we only trust that we shall not hear of any further experiments on living animals to make the assurance doubly sure.

This first article commemorates the establishment of a physiological truth; the next one records the vain attempt to prop up a pathological error. There can be no doubt that, in spite of the energetic attempts of some clever men in France, M. Broussais' system is manifestly on the decline, even in the land of its nativity. It may be all very well to try and give an *eclat* to it by addressing pompous eulogies to the memory of the "grand reformateur," but this alone will not do.

Like many other great *reformers* (!) of the present day, M. *Broussais* has been already weighed in the balance and found wanting. He strove to overthrow all the old and established usages and doctrines of his predecessors, and to substitute a system that, by its apparent simplicity, gained for a time a host of admirers and followers. He has been called, by some of his countrymen, the Napoleon of medicine!

In one respect only does the comparison hold good; neither has succeeded in establishing a dynasty that has endured.

The third article, a notice of M. *Louis*'s work on Typhoid Fever, may appropriately follow the description of M. *Broussais*'s inauguration. We have been surprised that some really talented physicians in this country have so much exaggerated the merits of M. *Louis*. As a pathologist, or, we should rather say, as a morbid anatomist, he is entitled to high praise; but as a scientific physician, or safe guide to follow in practice, he is below mediocrity. What value can we attach to the precepts of a man who has discovered that bleeding and blistering have little, if any, effect in subduing active inflammation of the lungs! M. *Louis* is one of those cold materialists in medicine, who can believe in nothing but what is revealed by the scalpel; and, as is usual with such men, he seems to be a sceptic on many points which are admitted as axioms by the bulk of his brethren. The remarks, which we have made in our notice of the second edition of his work on Typhoid Fever shew, we think, how vague and undecided his opinions are, even on a subject to which he has devoted the most assiduous attention, and on which he is considered to be quite an authority by many of his countrymen. We much fear that there will always be, in the French school of medicine, a large section of men who cannot stretch their views beyond the mere material phenomena of diseases, and whose ideas must therefore remain "cabined, cribbed, confined," within the limits prescribed by some favourite dogma. One reason for this state of things in the present day is the lamentable ignorance, which prevails among them, of foreign medical literature. They are nearly in the situation of persons who have never left their native town or district, and who very naturally suppose that *their* habits, and usages, and opinions are necessarily the right ones, and that whatever differs from them must be the result of inferior intelligence and refinement.

Although this charge is true of French authors generally, there are some very honourable exceptions to it among the physicians of Paris; and, in a recent number of this Review, we mentioned the name of M. *Rayer* with especial praise, as a man of extensive erudition and fine scholarship.

With the view of shewing that a counter-current is beginning to make head even in France against the stream of Broussaisism, and his *localising* doctrines, we have added one other extract from the recent lectures of M. *Andral* on the Changes of the Blood in Disease. In our late numbers we have so largely dwelt upon this subject, that it is unnecessary to say more at present than to recommend our readers to watch the development, or perhaps we should rather say the resuscitation in an improved form, of a system which will ere long overthrow the tottering edifice of an exclusive *solidism*. It is fortunate that it has fallen to the lot of so consummate, and withal so unbiassed, a pathologist as the author of the Clinique Medicale and the Pathologie Generale, to take the initiatory step in the establishment of a sounder nosology; as no one can accuse him of being a visionary, or of being ignorant of the discoveries of modern times. If no other benefit resulted from these researches of M. *Andral* on the blood, than that of most convincingly shewing that there is an essential difference in the state of the vital fluid in the pyrexiæ from what exists in the phlegmasiæ, that alone would be sufficient to stamp them with high value—not that this is any novel discovery; far from it; it is the common doctrine of the leading physicians of last century. The great merit of M. *Andral* is in having recalled the attention of medical men to the fact, and in having established its truth by a series of exact experiments. Does not this shew the impolicy of our neglecting the study of the older authors, and of our hugging ourselves in the belief that the present state of medicine is so far ahead of what it ever was before?

The letter of M. *Double*, to which we have already alluded, comes very *à propos* at this time, and we solicit our readers' especial attention to the extracts which we have given from it, and also from the reply of M. *Fred. Dubois*—"who," we are informed by a writer in the Gazette Medicale, "is one of the few physicians in the present day who do not regard the study of literary and philosophical pursuits as incompatible with the more immediate requirements of our profession."

The short biographical sketch of the late M. *Richerand,* which follows, is not devoid of interest; it suggests more than one useful lesson to medical men. In the case of him, as well as in that of his more successful competitor, M. *Dupuytren,* we see the painful effects of an ill-regulated mind. Both were men of great professional talents, and yet neither achieved the good that they might have done. Both were unhappy in their lives: the one was the victim of envy and disappointment, the other of an unsatisfied ambition, and of a gnawing inward chagrin. How striking is the contrast between the close of their career, and that of their great countryman, Baron *Cuvier*; after a life of unsurpassed honour and distinction and success, how calm and dignified and religious was his end!

---

### On the Physiology of the Nervous System: confirmation of Sir C. Bell's Views.

We have been much pleased with the perusal of a candid review, in the Gazette Medicale, of a work recently published at Paris by M. *Longet,* and entitled "Experimental and Pathological Researches on the properties and functions of the bundles of the Spinal Marrow and the roots of the Spinal Nerves, with an historical and critical examination of the various experiments made upon these organs by Sir *Charles Bell,* and others, &c."

The great discovery of the English physiologist had of late years, we are told, began to be questioned, and even by some to be fairly denied; for we find that, at a Meeting of the Academy of Medicine, about two years ago, one of the members undertook to demonstrate that the doctrine of the difference of function in the roots of the spinal nerves was a mere hypothesis devoid of all solid foundation. This bold assertion was certainly combated by several of the gentlemen present; but the effects of the discussion that then took place was certainly to leave a general impression that the views of *Bell* had received a decided check, or at least that its weak side had been exposed. It was with the view of fairly setting the question at rest either one way or the other, that M. *Longet* undertook a series of experiments, which were afterwards repeated in the presence of such judges as MM. *Flourens, Blainville, Gerdy,* and *Blandin,* and the result of which has been, in the language of another distinguished member of the Academy, M. *Breschet,* "to give a mathematical demonstration of one of the most important facts in the whole range of physiological science." We (the Rev.) might say that this mathematical demonstration had been already afforded by the beautiful experiments of Müller, of Berlin, on frogs, the earliest notice of which in English medical literature appeared in the pages of this Journal.* The French have thought otherwise; and glad we are that the question at issue may now be con-

---

* Vide the Medico-Chirurgical Review, for January 1834. The interest and value of the paper in question are greatly enhanced by its having elicited from Sir *Charles Bell* himself an admirable letter addressed to Dr. *Johnson,* and which is published in the same number. It will amply repay the trouble of referring back to it, not only for the facts which it communicates, but also for the comments which the distinguished author makes on the French physiologists.

sidered as fairly settled even by them; for we cannot conceal our abhorrent dislike of what they call vivisections, in which unoffending brutes are made the victims of the most shocking sufferings, all with the view of advancing science! Rather perish science, we almost feel inclined to say, if it cannot be promoted save by outrageous cruelty; but, thank Heaven, there is but little need of such bloody experiments as are far too frequently resorted to by the medical men of the French school.* What a different example has been set by the great English physiologist of modern times, the author of that very discovery which we are at present considering—a discovery, the beauty and value of which are co-equal with that of *Harvey* himself.

The work of M. *Longet* is divided into three parts. The *first* is occupied with a minute critical examination of all the various experiments, which have been performed by different physiologists from the period of Sir *C. Bell's* first announcement of his views down to the present time.

"This review," says the writer in the *Gazette Medicale,* "will by many be considered severe; but no one can have a right to complain; for there is nothing personal or arbitrary in it; and if it appears at times somewhat rude, it is because facts speak for themselves, and their language is often anything but polite, as we all well know." In reference to the experiments made by several authors on the anterior and posterior roots of the spinal nerves, M. *Longet* disapproves entirely of the plan which has been usually followed—that of merely dividing one set of them in order to judge of the effect of such a division by the paralysis either of sensation or of motion which may ensue. "The laying open of the spinal canal, independently of every other mutilation, must greatly affect the motory power of the animal; and hence, if the operator then proceeds to divide the posterior roots, the loss of motion that *may probably be* observed proves nothing, for this good reason, that it was nearly gone already. He therefore gives a decided preference to the use of *irritation* (galvanic) over that of simple *section,* in judging of the effects of such experiments. The loss of sensation in any part will always, be it remembered, influence to a very considerable degree its powers of motion under the application of mechanical or chemical stimuli."

*Müller*, however, it must be remembered, has anticipated by several years this very remark of our author, as may be seen by referring back to the account which we gave of his interesting experiments on frogs in this Journal, for January 1834.

The *second* part of M. *Longet's* work is devoted to a critical narrative of the pathological arguments in favour of *Bell's* doctrine furnished by the histories of numerous recorded cases of disease. "Nothing," says the French reviewer, "can be more conclusive than the mass of facts thus spontaneously supplied by nature, and all converging to one point. The observations, however, being drawn from various published works, this circumstance, although it is a fresh guarantee of their authenticity, tends to deprive this portion of our author's volume of that original interest which attaches to the first and third portions. We shall only remark that, if the number of conclusive cases which he has brought together seems to be very small, this does not arise from his having designedly excluded any that appeared contradictory of his system, but solely from his unwillingness to admit any except those which contained a definite and well-marked result, whether for or against *Bell's* doctrine. Thus out of 350 recorded cases of diseases of the spinal-marrow which he has passed under review, he admits

---

* Shakespeare says, with no less truth than beauty, of Cymbeline's Queen —who was an amateur experimenter with "poisonous compounds" on animals!—

"Your Highness
Shall from this practice but make hard your heart:
Besides, the seeing these effects will be
Both noisome and infectious."

not more than 20 in which the lesion was ascertained on dissection to be limited to one of the bundles, or to one of the series of roots, and in which the symptoms during life had been prominently marked. We may add, that of these 20 cases one only has appeared to be at all opposed to the theory of *Bell*, and the details of this very case are so very imperfect that we cannot fairly regard it as a conclusive exception."

In the *third* section of his work M. *Longet* gives an excellent description of the numerous experiments and researches which are peculiar to himself, with the view of determining the functions of the fasciculi of the spinal-marrow, and of the anterior and posterior roots of its nerves. The number of the experiments which he has performed on dogs and other animals amounts to no fewer than 330, many of them too in the presence of the most competent, and sometimes even prejudiced, witnesses. The results of these experiments have been so uniformly in favour of *Bell's* views, that we may well say with M. *Breschet* that our author has given the *mathematical demonstration* of a fact which many physiologists obstinately continued to dispute, even after the experiments of *Müller*, *Panizza* and *Valentin* were known to the public. The almost unvarying uniformity in the results obtained by M. *Longet* can only be regarded as the consequences of an established law in the animal system.

The following is the manner in which he conducted his experiments—which, however conclusive they must be admitted to be, we (the Rev.) must again express our abhorrence at, as unnecessarily barbarous and uncalled for.

The lumbar portion of the spinal canal in a dog was laid open, the anterior and posterior roots of the nerves were divided transversely, and then the one root was separated from the other as far back as the ganglion which exists on the posterior one. This being done, the *peripheral* extremity of the anterior root was laid upon a plate of glass, and the two poles of a galvanic pile—consisting of 20 pairs of 4-inch plates—were applied to this extremity. The animal exhibited no indication of pain, but violent contractions of those muscles only which receive twigs from the divided root were induced.

When the posterior roots were treated in the same manner, not the slightest convulsive movement was observed to take place.

When the poles of the instrument were applied to the *central* end of an anterior root, no movement was induced; but when they were applied to the *central* end of a posterior one, all the parts of the body (and not only that part on which the nerve whose root had been divided is distributed) where thrown into the most violent convulsions—a fact which shews that they were owing to the extreme pain felt by the animal.

Similar phenomena, although in a less marked degree, were induced when a mechanical stimulus was applied in lieu of the galvanic.

As some physiologists have obtained results somewhat different from those now stated, it will be useful to allude to the causes of the errors that have been often committed in conducting such experiments. Without again alluding to the confusion that is always apt to follow the employment of an over-powerful galvanic pile, we may remind our readers that the nervous cords, being, like all moist bodies, conductors of galvanism, may transmit the galvanic fluid according to physical laws and contrarily to those of the living organisation. M. *Longet* has for example satisfied himself that, by placing the posterior root of one of the spinal nerves in communication with one pole of a battery and the muscles of the thigh with another, the whole limb is thrown into convulsions: in this case the motific irritation must have travelled along a nerve which is exclusively sensory. This result is observed whenever the energy of the battery is very considerable.

M. *Magendie* has asserted that the anterior roots are sensory (jouissent de la sensibilité). The experiments of M. *Longet* have been uniformly and decisively contradictory of this opinion. The discrepancy in the statements of the two physiologists may perhaps be accounted for in this way. In the dog, as in man, the lumbar or sacral nerves are sometimes observed to consist of three distinct cords; of which one belongs to the anterior and the other two to the posterior root. It may therefore very readily happen that, when an experimenter supposes that he is touching the anterior root, he may have got hold of the deep-seated cord of the posterior one, and thus that the phenomena of sensation only will be manifested, when he expected muscular contractions to be induced.

We deem it unnecessary to do more than merely to allude to the experiments described by M. *Longet* with the view of shewing that the anterior cords or bundles of the spinal-marrow itself are essentially *motific*, and the posterior ones essentially *sensific*. Suffice it to state that these experiments demonstrate—as satisfactorily at least as such mutilating experiments can do—the fact of the difference in function of the anterior and posterior roots of the spinal nerves.

M. *Longet* arranges the various nerves of the body in three classes—1, nerves of special sensation, the olfactory, the optic, and the auditory; 2, nerves of motion, viz. the anterior roots of the 31 spinal, seven cranial pairs, the motors of the eye, the masticators (motor roots of the trigemini), the facial, the hypoglossal and the accessory; and 3, nerves of general or common sensibility, viz. the posterior roots of the spinal, the trigemini, the glosso-pharyngeal, and the pneumogastric. This arrangement is very faulty in many respects; but we cannot at present discuss the subject.

Although by the combination of sensory and motory filaments in the same sheath, the greater number of nerves become in their course endowed with compound functions, it is no less true that there are no nerves which are *mixtes* (*i. e.* sensory and motory at the same time) at their point of union with the cerebro-spinal axis. M. *Longet* has satisfied himself of the truth of this assertion by numerous experiments. When any of the nerves, placed in the class of *motors*, were divided at their origin and galvanised, contractions of the parts on which the nerve is distributed were invariably induced; and, on the contrary, whenever any of the sensory nerves was so treated, the animal always manifested pain, but there were no muscular contractions.

To ensure success in these experiments, it should be remembered—1, that the nerves experimented upon must be carefully detached from the surrounding tissues; otherwise the galvanism may act on the adjacent cord; and 2, that the nerves must be taken at their points of emergence from the cerebro-spinal axis, before they have received any *alien* filaments. It has no doubt arisen from the neglect of these precautions that different results have been obtained by different physiologists from apparently similarly conducted experiments.

By keeping in mind the important fact that many, both of the sensory and motory nerves, receive in their course filaments of a kind different from their primary cords, we can explain various morbid phenomena which are otherwise obscure.

The sensibility of the facial is derived from its anastomoses with twigs of the fifth pair. The paralysis of the velum palati, a not unfrequent accompaniment of facial hemiplegia, is at once interpreted when we understand that the glosso-pharyngeal (a sensory nerve) receives an anastomotic filament from the portio dura. It must be admitted, however, that all the facts of this kind are not so easily explained; and it is often a difficult problem to determine, whether a communicating filament between two nerves of different functions proceed from the motory to the sensory nerve, or vice-versâ.

With respect to the great sympathetic nerve, M. *Longet* is of opinion, that each of its numerous ganglia receives from the spinal nerves both a motory and a sensory filament, and one or several twigs besides by which it communicates with the ganglia in its neighbourhood.

Before closing our notice of this valuable work, we must not omit to mention that our author has extended his researches to some of the invertebrate animals, and that he has obtained results which seem to prove that the same distinction exists between the two sets of nerves in them, as in animals of a higher grade. The comparative anatomist is aware that in the *Crustacea,* the nervous chain on each side consists of two separate longitudinal cords, of which one is superior, the other is inferior, and that it is only on the latter that there is any appearance of ganglia. This arrangement at once suggests the idea that the inferior (or dorsal) cords are sensory, and the superior are motory.

M. *Longet* performed some experiments upon lobsters, with the view of determining the effects of irritation on these two sets of cords. He found that, when he irritated the nervous roots which proceed from the superior cord, the animal exhibited no signs of suffering; but that it always struggled a great deal whenever he pricked with a lancet one of the ganglia on the inferior cord. When the inter-ganglionary cord was divided across, that part of the body, supplied with nerves below the point of section, became motionless. By thus irritating the superior face of the caudal end of this cord, contractions, although in a feeble degree, were observed to take place; whereas, there was not any appearance of such on irritating its inferior or ganglionary face. These experiments perfectly accord in their results with those already obtained by *Valentin,* and strongly confirm the accuracy of his conclusions.*—*Gazette Medicale.*

---

### Letter from Mr. Shaw on M. Longet's Work, with Remarks on the Mode of Conducting Physiological Observations.

We had recently an opportunity of conversing with Mr. *Shaw*—whose intimate knowledge of all, that has been done and written about the nervous system of late years, entitles his opinions to high consideration—on M. *Longet's* work, and we gladly avail ourselves of his permission to introduce a letter which he has addressed to us on the subject of the preceding article. Mr. *Shaw* says,—

"I have been acquainted for some time with M. *Longet's* book. The experiments, which he has related on the roots of the spinal nerves, have been performed with so much care, and the results have been so decisive, that it is to be hoped the profession will at length unite in manifesting a general feeling, that the repetition of experiments, attended with so much cruelty, is no longer excusable.

In reference to the attempts to solve any questions concerning the distinct endowments of the nerves by experiments on the lower animals, it has long appeared to me a subject of regret, that the notion should have obtained such a strong hold as it has done on the minds of physiologists, that this plan of investigation was so pre-eminently superior to that of drawing deductions from the anatomy. It will be remembered by many that, soon after the first promulgation of the improved views in reference to the functions of the nervous system, a school of physiologists, assuming to themselves the name of *experimental phy-*

---

* Sir *C. Bell,* in his letter to Dr. *Johnson* already alluded to, adverts to the confirmation of his views by comparative anatomy........... "When I had yesterday the pleasure of meeting you in consultation, I shewed you a preparation which I value more than all the contributions from experimenters. It is a dissection by Mr. *Newport* of the nerves of one of the articulata, in which the analogy of the great central cord with the spinal nerves of the vertebrata is made out. In one aspect of the cord, you see the nervous matter forming a succession of ganglions; on the other, you see a column of nervous substance running over these ganglions, in the whole length of the cord. Can there be any doubt that these different columns are for sensation and motion?"

*siologists,* sprung up. The professed tenets of that school were to withhold belief from every deduction drawn from anatomical structure; and to consider such deductions hypothetical and unproved, until they had been over and over again submitted to the test of experiments on living animals.

Good service would be done to the profession at the present day, if the influence of that class of physiologists were lessened. That object could not perhaps be so effectually gained, as by some one, competent to the task, showing how various questions which have given occasion to the performance of experiments without number—followed too often by conclusions of the most incongruous and contradictory nature—might be finally settled, by following the anatomy correctly, and keeping certain well-established principles in view.

To treat the subject properly, would require more ample illustration than there is at present room for. But the statement I have just made applies in an especial degree to the question of the distinct functions of the two different roots of the spinal nerves. The profession must be well persuaded, by this time, what a difficult task it is to obtain certain and uniform results by having recourse to experiments on these parts. No better proof is needed than the very circumstance of M. *Longet* deeming it necessary, thirty years after the experiments were performed for the first time in this country, and when they have been repeated times innumerable, to enter upon a new series, and publish an elaborate work on the subject.

It has always appeared to me that, if we examined the question by drawing inferences from the anatomical structure, and adopting, as our guides, certain acknowledged principles acquired from the same source, we might obtain conclusions, the truth of which the most sceptical could scarcely deny.

A few remarks will illustrate my meaning.

1. Reason alone suggests that a nerve of motion should be anatomically distinct from a nerve of sensation. As these influences travel in contrary directions—that which induces muscular action being propagated *outwardly* from the brain, and that which gives rise to sensation being conveyed *inwardly* to the brain—it surely appears highly probable, at least, that each property must belong to a distinct structure.

2. The ninth pair has long been considered a motor nerve. Let us enquire what has led former physiologists to form that opinion. This nerve supplies the tongue, an organ endowed with motion and with sensibility: but it is remarkable that it is limited in its distribution to the muscular substance alone, and that it avoids sending any of its branches to the surface, which is therefore supplied by a different nerve. Now this circumstance, viz. that the ninth pair is limited to the supplying of *muscles,* carries to my mind as distinct a proof that it is subservient to motion, and that it has been correctly called the motor of the tongue, as the origin and insertion of the biceps muscle of the arm do, that the action of its fibres is to bend the fore-arm on the arm.

3. If we observe the peculiar structure, and also the exact point of origin, of the ninth pair, we shall be convinced that it is in truth an anterior root of a spinal nerve. In no one character can we recognise a difference between them. In short, the ninth nerve is in every point of view like a spinal nerve, which is deficient in a posterior root. May we not therefore infer that the anterior roots of the spinal nerves have a similar function to that of the ninth pair: in other words that they are nerves of motion?

4. Guided by these views, which naturally lead us to the belief that the whole line of nerves from the ninth downwards, arising from the anterior column of the spinal-marrow, are subservient to motion, we look with natural curiosity to those nerves which originate from the same column prolonged into the brain.

The first nerve which meets us is the sixth pair, a nerve that goes to a muscle alone; the next is the third pair, which likewise goes to muscles exclusively. Now in the same manner as it was inferred from the distribution of the ninth pair, that its function was to confer motion, so it may be concluded that these two nerves are destined for the same purpose. Indeed the fact cannot be forgotten, that to one of them the name *motor oculi* has been applied for centuries past. The course of observation hitherto pursued consequently leads us to infer, that the whole series of nerves arising from the anterior column of the spinal-marrow, from the third of the brain to the lowest nerve of the cauda equina, is destined for the giving of motor power.

So much for the uses of the anterior roots; let us next briefly attend to those of the posterior roots.

5. The fifth cerebral or trigeminus nerve is chiefly remarkable, in its anatomical character, for having an exact resemblance to the spinal nerves. This resemblance, it may be remarked, attracted the attention of anatomists, for some time before the recent discoveries in the nervous system were thought of. The peculiar character, which distinguishes the fifth from all the other nerves of the brain, is that it originates by two roots,—one of which corresponds with the anterior roots of the spinal nerves in having no ganglion upon it, while the other root has a ganglion exactly similar in structure to that formed on the posterior roots of the spinal nerves. Our line of argument, therefore, leads us to enquire whether, in the anatomical arrangement of the roots of the 5th pair, anything presents itself which can throw light on their distinct endowments. Now there is a circumstance in the anatomy of this nerve which is greatly in favour of our obtaining the desired knowledge. Instead of both its roots being of equal dimensions, that, which has a ganglion, is about four times as large as that which is without one; and the former can therefore be traced to many parts of the head where it is not accompanied by the latter. If we consider the lesser root, the analogue of the anterior roots of the spinal nerves, in the first place, and trace it to its destination, we find that it is distributed exclusively to muscles—it is the nerve proper to the muscles of the jaws. Indeed *Paletta,* resting on the circumstance of this root not going exclusively to muscles, so far anticipated the present views in regard to the physiology of the 5th, as to call this root a motor nerve, and to represent it as the nerve on which *trismus* depended. Accordingly, the observation of the simple distribution of this smaller root of the 5th sanctions the conclusion that, inasmuch as it resembles the anterior roots of the spinal nerves, and, I may add, the 9th pair, as well as the 6th, and the 3rd, in its structure, it is, in all probability, identical with them in function—namely, in being a nerve of motion.

I proceed next to the larger root, which from having a ganglion resembles the posterior roots of the spinal nerves; and will endeavour to shew how far the distribution of its branches tends to elucidate the nature of its functions.

What principally strikes an anatomist, when surveying the course of the several great branches derived from this larger root, is that they supply numerous parts of the head where no muscles exist, and when there are only sensible surfaces. I will put aside as doubtlful the branches which emerge upon the face, since the muscles of the features appear to be supplied by these branches in common with the portio-dura.

But taking other branches, we find that several pass to localities where no muscles are situated, and where of course we cannot suppose that nerves of motion should be sent. For example, we have the cavities of the nose liberally supplied with branches proceeding from the ganglionic root; we have branches of considerable size from the same origin going to the hard palate: branches of the same kind go to the teeth: and, lastly, there is a large branch, the proper destination of which is manifestly the sensible surface of the tongue.

These facts, coupled with the circumstances that the whole extent of the integuments of the head, (except a part behind, which is supplied by spinal nerves), has branches of this root sent to them, afford by themselves strong presumptive evidence that the office of the ganglionic root of the fifth pair is to confer sensation as distinct from motion.

Now, if the inferences stated above be thought justly deducible from the premises, they may be applied legitimately to the explanation of the functions possessed by the posterior roots of the spinal nerves. We are thus led to conclude that, as the function of the larger or ganglionic root of the fifth pair is to bestow sensation, so it is the function of the posterior or ganglionic roots of the spinal nerves also to bestow sensation.

In this simple manner have we been brought, without the performance of a single experiment on a brute creature, but by relying exclusively on anatomy as our guide, to educe the beautiful truth, that the anterior roots of the spinal nerves, including their analogues in the encephalon, are subservient to motion, and the posterior roots of the spinal nerves, together with the ganglionic root of the trigeminus, to sensation.

It cannot be questioned, then, that those physiologists deprive themselves of a valuable instrument in the prosecution of the inquiry, who, placing too great confidence in the plan of experimenting, refuse to take advantage of the mode of investigation which I have endeavoured briefly to illustrate. With reference to the roots of the spinal nerves, it is scarcely too much to say, that, had a course of demonstration similar to what I have here imperfectly sketched out, alone been followed, and if physiologists had waited patiently till cases occurred in practice —such as have actually been met with in very numerous instances—where the pathological phenomena confirmed the views deduced from anatomy, our convictions as to their distinct offices would have been as strong, as they are at this moment, after all the multiplied experiments which have been performed.

Yours truly,

ALEX. SHAW.

23, Henrietta Street, Cavendish Square.
25th January, 1842.

---

### INAUGURATION OF THE STATUE OF BROUSSAIS.

There was something so truly national in all the "pomp and circumstance" of this grand solemnity, celebrated on the 21st of August last at the Military Hospital of the Val de Grace in Paris, that we are tempted to quote rather largely from the full report that was published at the time. Its exordium is *un peu* magniloquent.

"Three years have elapsed since the death of *Broussais*. The first of December 1838 will ever be regarded as a great historical date. On that day science was struck in the head; that day beheld the extinction of one of the brightest intelligencies that have ever illumined the world; the nineteenth century suffered an irreparable loss, and France wept o'er one of her most glorious children."

The funeral, we are told, was truly grand; the hearse was transformed into a car of triumph; the procession through the streets of Paris occupied upwards of five hours; the great capital was awe-struck, and for a moment dumb with grief. Who can forget the moment when the bier stopped in the Place Vendome under the eyes of that other genius that hovers on the summit of its pillar of brass? There was something sublime in this salutation of the mighty dead!

In the hall of instruction at the Val de Grace, where the statue was erected, three large and decorated tents had been put up for the accommodation of the visitors, one being reserved for the ladies. In the centre was a desk or tribune for the orators who were to address the assembly. Every place was occupied long before the hour appointed for the commencement of the imposing ceremony.

Groupes of soldiers were arranged round the tents, and the musicians had taken their seats. At two o'clock precisely, the various members of the commission arrived to the sound of trumpets, headed by M. *Orfila*, the president, in his crimson robes. Among the most conspicuous of the deputation was the noble and illustrious *Larrey*, who seemed to wear on his brow that magnificent appellation, "homme monument," applied to him by the emperor on his death-bed. "The moment of most intense interest has now arrived; anxiety is painted on every countenance; the trumpets are blown; and the veil, which had hitherto concealed the statue, is withdrawn. All the assembly rise at one instant; and, with clapping of hands, exclaim, "Voila Broussais!" There he is at home, in his own Val de Grace, among his friends, surrounded by his disciples and his children. He is seated in his arm-chair, attired in his robe-de-chambre, long trowsers, and slippers, such as we have so often seen him in his study." Our thoughts were involuntarily carried back to the time of our medical studies; we remembered the patient visit of the great physician; we saw him stop at every bed; we heard his voice ever mild and encouraging to his patients—for this man, terrible although he was in intellectual conflicts, this truly formidable dialectician, had his moments of most winning and almost infantine sweetness.

At times indeed, in the presence of a case, which demonstrated the truth of his doctrines and the perverse errors of his adversaries, his voice would rise, his mouth would assume that indescribable expression, in which anger, irony and contempt were blended together, and the patients would raise themselves up in bed to contemplate this man, who in their eyes seemed at the time a strange and almost superhuman being. His walk was quick, almost violent, but always dignified and sometimes majestic. When he wrote there was the same energy in his movements as when he spoke; the paper "criait" under his pen, held, as it was, in a hand of iron.

The first who addressed the assembly was M. *Passy*, the vice-president of the Academy of Moral and Political Sciences. He enumerated in a simple but affecting style the numerous claims of the illustrious deceased to public gratitude and admiration, and pointed out the share which he had so often taken in our recent philosophical disputes.

M. *Pariset*, the perpetual secretary of the Academy of Medicine, followed. His oration was certainly not such as was expected from so eloquent a speaker. The task seemed to have been any thing but a willing one; for, at the very commencement of his equivocal eulogy, he repeatedly spoke of *fulfilling a duty*. The greater part of his discourse was a dissertation on the immortality of the soul; and when, in the very face of the man whose death he rather burlesqued than deplored, he had the hardihood to say that *Broussais*, in his last hours, had acknowledged the truth of revelation, signs of disapprobation began to be expressed by the audience. M. *Pariset*, although an able man in his way, is a weak timorous religionist, and was perhaps afraid of calling down upon his own head the thunders of the church for giving his public approval of an act, which may not be regarded as sufficiently orthodox by the ministers of religion.*

---

* We know not why it should be, but the fact cannot be well denied, that most of the prominent advocates of phrenology have been and still are very lax in their religious principles. *Spurzheim* was a cold and doubting believer; *Broussais* was an utter sceptic; and M. *Bouillaud* seems to wear his religion very loosely. Alluding in the Academy to a recent visit at a large school, the pupils of which, after a due examination of their heads, were arranged in two groupes, one on the right side and the other on the left of the phrenological inspector, he said that the exhibition reminded him of the description of the day of judgment in Holy Writ; and that the comparison was the more striking from the circumstance that "many were called, but few were chosen"—the criminal develop-

M. *Bouillaud* then mounted the tribune as the representative of the faculty; and never was the inspiration of this ardent professor more lively or more attractive. He was deeply affected; his austere visage wore the expression of a feeling at once sad and triumphant. The recollection of a departed master and friend depressed, while the glory of the apotheosis elevated, his mind.

"Broussais!" he exclaimed, "thy day of judgment hath now come: and when I here solemnly proclaim that you were a man of genius, I feel confident that every listener will respond to my words, and that posterity will confirm their truth. Yes, it was you who laid the edifice of medicine on its proper foundation, and erected it upon the basis of anatomy and physiology. You have shed over the whole domain of our science the rays of the clearest light, by substituting for what you so happily designated 'ontology' the physiological doctrine of medicine, and by completing the work of the great *Bichat*. You have revealed one of the most important truths of medicine by demonstrating that essential fevers are only disguised inflammations; and you have conferred the most signal benefit on humanity by substituting the antiphlogistic for the stimulant and incendiary method of treatment in the cure of those monsters (to borrow your own energetic language) known by the name of ataxic, adynamic, and malignant symptoms. You have revealed another important truth—that diseases are not necessarily subject to the course and duration which have been assigned to them by your predecessors, but that these points in their history are chiefly dependent upon the mode and the energy of the treatment employed. (We suppose that this remark alludes to pyrexiæ in general, and more especially to the groupe of the exanthemata.) In fine, you have created a theory as just as it is ingenious and brilliant, tracing to a process of chronic inflammation a multitude of lesions which have been termed organic, and which before your time were considered as diseases *sui generis*, and independent of all inflammatory action."

Alluding to the fine statue before them, M. *Bouillaud* continued:—"There stands the great reformer of medicine in all the beauty and grandeur of his expression, in all his force, in all his vigour, in all his energy, in all his severity, and, if I may venture to say so, in all his hardy, resolute, and even threatening mien. There stands *Broussais* alive, his head filled with high meditations, with deep thoughts, with revolutionary enterprises; trampling under his Herculean foot those works of ontology on which he had passed so signal a condemnation. His look is bold, imperious, and confident; his mouth is slightly contracted, his upper lip quivering with that peculiar movement which was so characteristic of the great original. Listen, for it seems as if this statue, like that of Memnon, was about to give out an oracular sound."........ "However, true honour to the *Phidias* who has represented so admirably the hero of this solemnity. The statue is truly worthy of the personage, and what can be said more in its praise? There is only one monument that can be more durable and glorious; and that it is the works of *Broussais* himself."

When M. *Bouillaud*, pointing to the statue of his master, uttered with his thrilling voice these "grand paroles," the whole assembly was visibly affected with "une emotion immense." The orator sat down among the most rapturous applauses, which drowned for a moment the sound of the trumpets!

---

ments being good only in a small number! (Are the British phrenologists an exception to the preceding remarks? we fear not. Witness some of their recent works, medical as well as literary.)

No wonder that the novels and light literature of France in the present day should be often so defiled with blasphemy, when even scientific discussions are not exempt from its taint. One of their journalists the other day spoke of Napoleon as being "Christ armé!!"

M. *Begin,* in the name of the military physicians and surgeons, then addressed the audience. It might have been expected that their attention would be rather exhausted by this time, three orations having been already pronounced; but this was so far from being the case that his discourse may be regarded as quite a triumph to his professional brethren of the army. In the latter part of his "somptueux panegyrique," he thus nobly vindicated the claims of *Broussais* to the admiration of posterity:—

"At the time of its appearance, his work entitled 'Examen des Doctrines Medicales' was regarded as an enormous reproach to the profession—the press was then afraid of having anything to do with it, and it would perhaps have sunk into oblivion at once, in the coalition of silence that was formed against it, if some young men, who, like *Broussais,* had recently returned from the army, had not seized upon it and proclaimed to Europe the prodigious grasp of the ideas which it contained. These youthful apostles of the new religion had neither the power of thought, the loftiness of the conceptions, the richness of the reasoning, nor the treasures of the experience of their great master; but their enthusiasm was kindled by the fire of his discourses; they were led on by the love of truth; no obstacle alarmed them; and they were animated with the belief that they might aid the giant in moving the world.

A multitude of chronic diseases, the origin and true nature of which had been either completely unknown or greatly misunderstood, were traced to the principle of irritation. This principle, vigorously defined and illustrated in its various modes of action, was shewn to be the main key of the medical edifice.

The symptoms of diseases no longer served for the purposes of classification, but were studied with the view of discovering what organs were affected, and of determining the nature of their morbid state—a mode of proceeding which overthrew the various nosologies which had been based upon mere external phenomena. Hence the physician, in the selection of his remedies, came to be guided, not by the apparent strength or weakness of the patient, but by the real condition of the diseased organs.

The occult principles, the characters of diseases independently of the organs affected, and all the phantasmagoria of ontology were exposed, attacked, and destroyed.

One entire class of diseases, perhaps the most numerous and important of all to which mankind are subject, that of essential fevers, comprehending epidemic and endemic pestilences, has been effaced from the nosological catalogue, studied in all its parts, and grouped among the inflammatory lesions of different organs.

The phlegmasiæ of the stomach and bowels were described for the first time, in a variety of shades, with so much exactness that it seemed as if they had never been known to medical men before.

Everywhere anatomy was made to serve as the basis of physiology, and physiology was appealed to to explain diseased functions; thus the study of actual facts was substituted for the reveries and illusions which had so long prevailed in almost every branch of medical science.

The reform which this great man has introduced into therapeutics, has not been sufficiently appreciated. Without rejecting really efficacious remedies, it was he who struck with the anathema of his denunciation the incendiary abuse of stimulants, and that absurd routine which prescribed evacuants at the commencement of many acute diseases. He had no faith, it is true, in certain formulas, nor in the use of any panacea or specific remedy. He did not look upon the stomach as an inert vestibule from which the various substances, which the physician might choose to introduce into it, could find their way by some special election to their wished-for destination; he did not believe in the occult virtues, or in the medicative powers ascribed to many of the articles of the Pharmacopœia; but he pointed out the necessity of watching the real impressions which they produce on different organs, and of ascertaining their local effects, their secondary influences, and at length their curative properties. Now true science will always follow such a course, and we cannot lose sight of these principles without at once yielding the place to empiricism."

After the close of M. *Begin*'s discourse, which was received with frequent acclamations of applause, M. *Fossati*, the president of the Paris Phrenological Society, rose to address the audience. He mentioned that, in 1829, the year in which *Gall* died, *Broussais* was not a phrenologist: "it is not one of the least titles of his glory, as it is not one of the least proofs of his genius, that, in the full maturity of his age, and when occupied with his multifarious professional pursuits, he entered upon the study of a new science, and pursued it with so much diligence and success, that he soon enlarged its domain."

The close of M. *Fossati*'s oration was singularly happy. Addressing himself to the students in the hall, he exclaimed, "It is not for *Broussais*, it is for you that this statue has been raised. It is less as an homage to him, than as an example for you." These beautiful words were rapturously cheered.

The whole assembly then rose, and individually approached the statue, thus closing, by an unanimous expression of homage, this "magnifique ovation."—*Gazette des Hôpitaux.*

*Remarks.*—It is not likely that the custom of pronouncing "eloges" over the graves of distinguished characters, or when any public monument is erected to their memory, will ever be adopted in this country. There is an admixture of the theatric display of modern times, and of the superstitious absurdities of the old classical ages, in such ceremonies, that does not recommend them to our ideas of what is either right or impressive.

The moment of committing a fellow-creature to the dust is rather too solemn and humbling to listen to pompous declamations on his greatness and glory: we prefer the simple and touching eloquence of our Liturgy. But the French view the matter in another light.

They are especially fond of imitating the Greek and Roman usages on all occasions when they wish to be more than usually grand. Their tragic literature is almost all "classique;" so is their school of painting. They have their Pantheon; they have their Temple of Glory; they have their Elysian Fields, &c. &c. and it is, therefore, quite natural that the funeral obsequies of their great men should be "after the high Roman fashion." Unfortunately they attach so little importance to moral integrity or to practical religion, that those graces of character are seldom or never alluded to in their panegyrical discourses; and we have seen, in the case of the inauguration of *Broussais*'s statue, that if any one should be so bold as to make reference to such matters, he is at once checked by the disapprobation of the auditors, who naturally wish to keep all the defects in the character of their idol in the shade. *Broussais* was both an immoral and an irreligious character. He was a materialist in his belief, and a shameless libertine in his conduct. Even as a man of science and a physician, his example is by no means one worthy of very high eulogy.

In spite of all that MM. *Bouillaud* and *Begin* and other disciples of the "great reformer," may say to the contrary, it is too obvious that, even in France, the edifice of the Broussaian doctrine is rapidly beginning to pass away, and is destined soon to share the same fate with all other exclusive theories in medical science. How *Broussais* can be regarded by any one as the legitimate successor of *Bichat*, who declared that "every exclusive doctrine whether of solidism or of humorism is a pathological absurdity," puzzles us to comprehend. That he has done good to medicine in his day we are far from disputing; and we readily admit that, by directing the attention of the physician to the important part which chronic inflammation plays in the establishment of many organic diseases, he has contributed to introduce a rational mode of opposing their development and progress.

On the other hand, a vast deal of error and positive mischief has been the result of his dogmatical explanation of the phenomena of fevers. The constant burden of his song was "gastro-enterite;" and many, we suppose, were induced to assent to it only from the ceaseless dinning into their ears of this far-famed word. Medical men are surely the most credulous mortals in the world. How they could bring themselves to believe that all the phenomena of typhus fever are traceable to a slight inflammation of the mucous coat of the stomach and bowels, and this, too, even when perhaps not one symptom of such an inflammation is present, may well surprise the most ignorant; and yet, in the present day, there are not wanting men of good education and experience who continue to vaunt this as one of the grandest discoveries of modern times. Little is the wonder that the treatment of fever is so ill understood, as it certainly is, more especially among the physicians of the French school. One teacher recommends the use of brisk purgation, and another condemns the practice; one continues to bleed all his patients, and boldly tells you that almost every one of them recovers, but, when you come to examine his statements, you probably find that more than one half of his cases of alleged typhus seem to have been nothing but instances of feverish malaise from cold and fatigue; a third gives frequent doses of chloride of lime; while a fourth does nothing at all, and, by merely "waiting upon Providence," succeeds quite as well as his more unbelieving brethren, who must ever be up and doing. We do sincerely hope that the admirable researches of M. *Andral,* on the state of the blood in various acute diseases, may soon have the effect of introducing a better state of things into therapeutical science. They are well calculated to do so; and, however humbilng it may be to the pride of the nineteenth century, to find that our improved means of scientific analysis are tendirg to bring back many of the long-exploded doctrines of our ancestors, while they expose the fallacy of more recent speculations, the sooner we shake off this pride the better. For our own sakes we have been so long impressed with the truth of many of the principles of a humoral pathology, that we gladly hail, and shall strive to second, the efforts of M. *Andral* to introduce among his countrymen more correct views on the effects of the changes which the blood undergoes in disease; for we feel confident that, while the so-called physiological doctrine of fevers must soon be committed to the tomb of all the Capulets, that, which is based on an attentive examination of both the fluids and the solids, is the only probable and truly satisfactory one. —(*Rev.*)

---

### M. Louis on Typhoid Fever.

We observe by the French journals that a new edition of M. *Louis*' well-known work on this subject has recently been published in Paris. Its title deserves notice: *Recherches Anatomiques, Pathologiques et Therapeutiques sur la Maladie connue sous les Noms de Fievre Typhoide, Putride, Adynamique, Ataxique, Bilieuse, Muqueuse, Gastro-enterite, Enterite Folliculeuse, Dothinenterie, &c. comparée avec les Maladies Aigues les plus ordinaires!* Does not this alone indicate how bewildered the ideas of medical men must still be on the all important subject of fever? We had almost hoped that M. *Louis* would by this time have found reason to modify his opinion as to the seat or proximate cause of typhoid fever being in the intestinal canal; but we find that we are mistaken. He still maintains, and even in more decided terms than ever, that "the anatomical character of the typhoid affection (a very stupid phrase) consists in a morbid alteration of the intestinal, chiefly the Peyerian, glands; and, also, that any secondary lesions are very usually of an inflammatory nature."

This doctrine is a most fallacious, and unfortunately at the same time a most hurtful, one. While we admit that the intestinal lesions seem, from all accounts, to be of much more frequent occurrence in fever as it occurs in France, than as it is seen in this country, we most confidently deny that they are uniform or constant even in the former case—at least if we can trust the testimony of such men as *Chomel* and *Andral*. But even admitting the frequency of intestinal lesions in fever, does it necessarily follow that they are the consequences of simple inflammatory action, as asserted by *Broussais*, *Louis*, *Bouillaud*, and others of this school? Do we observe the same phenomena on dissection in typhus as are found in fatal cases of genuine enteritis? Assuredly not. The patchy redness in different parts of the canal, the tumefied and softened condition of the mucous and submucous tissues, the enlargement, ulceration and even sphacelation of the follicular glands, &c. cannot surely be admitted as the legitimate evidences of a pure phlegmasia. They might rather be regarded as the results of the irritation of unhealthy acrid secretion on the mucous surface than as the primary and essential phenomena of the disease. But without going so far as this, we have no hesitation in asserting that the intestinal lesions, however frequent they may be in the fevers of Paris, can only be viewed as one of the several changes induced by the general disease of the system. To apply the term of *enteritis* to such lesions is not only incorrect in theory, but most pernicious in practice. It has been from the too general adoption of this dogma by the present generation of medical men in France, that so many and so serious errors have been committed by them in the treatment of their patients. When we read the grave discussions that are frequently agitated in the Academy of Medicine of Paris, on the results of some novel mode of treating typhoid fever, what are we to expect from the young surgeons and physicians who are annually drafted away either to the country, or perhaps into the army and navy, immediately after having had their heads crammed with the "nouvelle doctrine" as taught by the professors of the physiological school!

Part of M. *Louis*' work is taken up with shewing that "jail-typhus and typhoid fever are identically the same disease." What rational man ever doubted it? But such are the vague opinions of French medical men on the subject of fever, that M. *Louis* deems it necessary to devote an entire chapter to prove a position, which has been recognised as an acknowledged truth by British practitioners ever since, and indeed long before, the days of *Huxham*. But then it is at the same time well known that almost every epidemic of fever, whether it appears in jails or other crowded buildings, or in the lanes and dirty alleys of a city, exhibits some peculiarity or another by which it may be characterised; and yet no sensible physician would dream of designating one epidemic as cephalitis, another as bronchitis, a third as gastro-enteritis, merely because the predominant symptoms may be at different times seated in the head, chest, or abdomen. Until medical men can be induced to carry their thoughts beyond the more obvious lesions discoverable on the dissection of patients who fall a victim to typhus fever, they will ever remain involved in a labyrinth of perplexities, which will only be increased by a more extensive acquaintance with the disease. A calm unprejudiced examination of the history of typhus must lead every one, we are inclined to believe, to the conviction that it is the result of an invisible atmospheric agent—call it miasm, malaria, matter of contagion, or what you will—on the human body, acting primarily on the nervous system, and often rapidly absorbed into the system, thereby inducing a more or less decidedly vitiated state of the circulating fluids.

The lesions of the viscera, whether of the cranial, or of the thoracic or abdominal cavities, are all secondary or consecutive phenomena of the disease: their occurrence being attributable either to the cotemporaneous influence of other atmospheric agencies, to the idiosyncrasy, habits, and previous state of health of the patients affected, or perhaps to the differences in the nature and degree of the aerial poison that has been introduced into the system. We must admit that, in taking this view of the question, there are many points that cannot be demonstrated by actual proofs, and that the theory is essentially based upon a mere probability; but let us remember that there is, and ever will be, much in the history of diseases, especially those of an epidemic and diffusive character, that must defy the scrutiny of human observation: we cannot *exhibit* the infectious poison of scarlet fever, or of measles, or the miasms which give rise to intermittent or remittent fevers; and yet no one disputes their existence. It is therefore a very insufficient reply to be told that we cannot demonstrate our theory of the disease. Some modern French writers are constantly trying to make us believe that medicine is capable of being rendered an *exact* science, and that we must not admit any position in our reasonings on disease but what can be proved by actual observation. This is a most foolish and dangerous doctrine. As in moral and political philosophy, so in medicine, there are no permanently fixed and unalterable rules which are inevitably true at all times and under all circumstances. The beauty of a moral precept or the expediency of a political maxim may charm the mind in theoretical speculation; but in our practical dealings with the world, we find, alas! that the line of conduct to be adopted must greatly depend upon the circumstances of each case—ever striving however to act on the principle of doing unto others as we wish them to do unto ourselves.

Dr. *Landouzy* of Rheims, the reviewer of M. *Louis*' work in the French Medical Gazette, states that his own experience in the late epidemic of jail-typhus in that town confirms the opinion that it is analogous, if not identical, in nature with the typhoid fever of the metropolis, and he bases his opinion on the circumstance of the *constant* existence, in all cases and at all periods of the disease, of the intestinal lesions in both forms of the fever. Yet, strange to say, he informs us that in no case were there any symptoms of such lesions during the life: "la diarrhée, la douleur abdominale, le meteorisme et le gargouillement ne se sont jamais rencontrés." How can Dr. *Landouzy*, or any other sensible man, in the face of such a statement as this, continue in the belief that the disease, in which these symptoms were absent, was an inflammatory affection of the mucous coat of the bowels; in other words, that it was a "gastro-enterite," or "dothinenterie." He suggests, as a novel idea, that the difference in the symptoms of typhus fever in different times and seasons may be connected with some difference in the type or genus, as he calls it, of the disease, and very coolly remarks that "the observations of authors on this point of pathology having in truth but little or no value until the 'beaux travaux' of M. *Louis* have become generally known, we must wait for new epidemics of typhus before we can offer a positive opinion upon the subject."

Has Dr. *Landouzy* never heard of *Sydenham* or *Huxham*, or of *Cullen*, or of his own countryman *Pinel*, not to mention a century of other authors, who have all insisted upon this essential fact connected with the history of fevers—the differences in the character of the disease according to what has been demonstrated the "medical constitution" of the season? Little is the wonder that the French are making discoveries in medicine almost everyday: they seem to be utterly unaware of their own well-expressed motto, "nothing is so new as that which is forgot!"

The therapeutic portion of this edition of M. *Louis*' work has received, we are informed, "d'immenses developpemens;" but the reviewer does not tell us particulars; all that he says is, that "the author *seems* to prefer in the greater number of cases the use of evacuants to any other remedies." The little word *seems* (semble) implies, if we are not much mistaken, a mighty deal in reference to M. *Louis*' mode of treatment. It is well known that this gentleman, however high he may be regarded as a morbid anatomist, cannot be appealed to as an authority on practical subjects. Some years ago, as we have noticed in a former article, he made the important discovery that bloodletting and blistering had little or no efficacy in subduing pneumonia! He has probably extended the application of this discovery to the treatment of fevers, and may have found out that by far the best mode of managing them is to do nothing.

In this respect his example offers a marked contrast with that of M. *Bouillaud*, a disciple of the same pathological school, whose "grande decouverte" is the "nouvelle formule" of bleeding "coup sur coup," so as to "juguler" the disease at once!! But we trust that it is quite unnecessary to expose the fallacy of this gentleman's views at present, as we have so repeatedly of late years cautioned the English reader against the errors into which he is apt to lead the inexperienced by the bold and confident manner with which he proclaims his marvellous success.

---

### M. ANDRAL ON THE CHANGES OF THE BLOOD IN DISEASE.

To complete our account of this eminent pathologist's recent researches on this highly interesting subject, we have extracted his observations on the different temperaments of the human body, as they are observed in different individuals.

A medical man should be well acquainted with this point, as the success of his treatment in a variety of diseases will much depend on the adaptation of his remedies to the peculiar constitution of each patient. The subject deserves the attention of the moral philosopher likewise in his appreciation of human character; but we cannot enter into this question at present, although it is far from being foreign to the pursuits of the scientific physician.

*Sanguineous Temperament.*—According to the researches of MM. *Andral* and *Gavarret* it would seem that the common notion that the blood in persons of this temperament contains a larger quantity than usual of its solid or fibrinous constituent is not strictly correct. There is but little increase in the proportion of this element; whereas that of the red globules is generally very considerably augmented—from 127 to 135 or 140 parts in 1000. If we examine the blood of such persons before it coagulates, it is found to be of an extremely bright red colour. The clot is usually large, from the retention of a large quantity of the serum in its meshes; but its consistence is not greater than in health; and often it is even less. One of the chief characters of this blood is that *it very rarely exhibits a perfect buffy coat*—although the very contrary opinion is so generally asserted. This circumstance is owing to the small proportion of the fibrine in comparison with that of the globules, which, as we have said, are predominant.

In persons of the sanguineous temperament, all the functions of the body are usually performed with great activity; life, so to speak, is in excess; and this excess is proportionate to the increase of the red globules. The digestive process goes on rapidly and efficiently; the respiratory apparatus is largely developed; and the minute capillary vessels are always more highly injected than in persons of other temperaments. The heat of the body is high; the perspiration is easy; and there is an abundant secretion of deep-coloured urine, which usually contains a large quantity of saline matter. The energies of the brain are easily exalted; the passions are quick, and vehement; and yet the sensibility is by no means excessive, and never so acute as in persons of the nervous temperament. Hence the class of diseases known by the term *neuroses* is not common in plethoric individuals. It would seem that, as the proportion of the red globules increases, so the sensibility of the system diminishes, and *vice versâ.* An extreme and morbid acuteness of the nervous system is well known to be a characteristic symptom of chlorosis and anæmia.

The pathological conditions of most frequent occurrence in plethora are congestion, hæmorrhage, and fever. Genuine inflammation is much less frequent; we might even assert—in opposition indeed to the generally received opinion—

that *plethoric persons are not more liable to the phlegmasiæ than those of other temperaments.* From the very circumstance of the globules being in an increased proportion, the normal relation between the proportion of this element and that of the fibrine is disturbed, and the consistence of the blood is rendered actually less than it is in health. Blood-letting, from its marked influence in reducing the proportion of the globules, always speedily relieves the diseases arising from plethora: its influeence on the fibrine is much less decided and speedy.

*The Lymphatic Temperament and Anæmia.*—The general fact to be mentioned in reference to this temperament is the diminution of the physical forces, and an enfeebled state of many of the functions of the body. There is usually a strong disposition to the development of scrofulous disease. M. *Lecanu* has stated, and M. *Andral* confirms the opinion, that the proportion of the red globules is always low in persons of this temperament. The surface is usually pale and puffy; the iris exhibits a more than ordinary clear hue; and the pilous system is but little developed. When any phlegmasia occurs in scrofulous persons, it proceeds in its course slowly, and less readily to resolution—a termination which is always long of being fairly established, and is apt to be interrupted by a variety of accidents.

The lymphatic temperament, when much exaggerated, leads, as we have already said, to the development of scrofula; the anæmic to that of chlorosis. In spontaneous or idiopathic anæmia, the proportion of the fibrine is often but little diminished; but, in that produced by great hæmorrhages, the blood is always deficient in this element. The proportion of the globules is sometimes remarkably diminished; it has been known to fall from 127—the normal standard—down to 40, and even as low as 27.

Although all the physical functions suffer, whenever there is any considerable diminution of the red globules the energies of the brain are often surprisingly little affected; the sensory functions are usually exalted; and the activity of the mind is not unfrequently increased. But the digestion is always more or less disturbed, and the patient often suffers exceedingly from one of the most severe and intractable forms of gastrodynia,* as well as from headaches and confusion of the sight, and of the other especial sensations. The actions of the heart and lungs, too, are very frequently more or less severely deranged. Every practical physician is well aware how much anæmic patients usually suffer from violent palpitations, and that the tictac of the heart is often accompanied with a blowing sound, such as is heard in cases of contraction of the arterial orifices. The blowing sound may be either constant, or it may be intermittent. The smaller that the proportion of the red globules is in the blood, the more constant this abnormal sound generally is.

It must, however, be admitted, that exceptions are sometimes met with to this assertion; and that, in some rare instances, where there has been no deficiency of the red globules, and no existing organic disease of the orifices of the heart, the blowing sound has been heard.. . . . . . . . . . . .

---

* Change of air and the use of steel are unquestionably the best remedies against this form of the disease in most instances. We recently had a very interesting case, occurring in a delicate, pale, phlegmatic woman, of about 33 years of age. She had been subject to the complaint for *several years,* with occasional intermissions, as a matter of course. Anodynes, bitters, alkalis, blisters and a host of other remedies had all failed Carbonate of iron was freely given in large doses; but without success. We then ordered the *genuine* steel wine—prepared by digesting two ounces of the filings in a bottle of the best sherry—in doses of an ounce three times daily. The benefit derived was very speedily marked: and the relief has now continued for four or five months.—(*Rev.*)

*Of the Blood in Fevers.*—We do not mean to recapitulate the details of M. *Andral's* numerous experiments on this interesting subject of humoral pathology, as we have dwelt at sufficient length upon it in the three last numbers of this Journal. All that we shall do at present is to pick out a few of his most interesting remarks, with the view of shewing how convincingly the study of the changes of the blood in disease demonstrate the fallacy of those opinions which have so long reigned paramount in the French school of medicine. It is fortunate for M. *Broussais* that he has not been spared the chagrin of seeing his favorite edifice crumble before the well-laid approaches of his great rival. We have been looking for the last twelvemonths to discover what M. *Bouillaud* and the other zealous advocates of the so-called physiological doctrine say of these doings in the French metropolis; but not a word is uttered, or at least made public. Are they reserving all their strength for some grand attack after the lectures of *Andral* have been completed? or is it really the case that they feel their ground untenable, and like wise generals are securing their retreat in silence without unnecessarily provoking the attacks of the advancing army?

The following brief paragraph will shew how completely their very centre has been pierced through in the conflict that has of late taken place. After alluding to the division of fevers into those that are idiopathic, and those that are merely symptomatic, M. *Andral* says: "Idiopathic fever may exist, 1, without any appreciable lesion of the solids, or 2, with a well-characterised lesion, but which however is not the cause of the disease. The carbunculous tumors and bubos in the plague, and the ulceration of the intestinal glands in typhus are, in my opinion, the effects of a cause which to me is yet unknown."

When a fever is symptomatic of a genuine inflammation, the proportion of the fibrine in the blood is increased; but when it is not, there is no increase of this element. Now the quantity of the fibrine is certainly not increased in typhus, unless indeed an accidental inflammation of some internal organ supervenes during the progress of the disease.

In reference to the cutaneous phlegmasiæ of eruptive fevers, M. *Andral* remarks:—

"A fever may shew itself at the same time as certain inflammations: these cannot be said either to have preceded or to have complicated it; they were generated with it, and constitute one of the essential elements of the disease. Of this nature are the phlegmasiæ of the skin in small-pox, scarlet fever, and measles; these lesions, *which are perhaps erroneously regarded as inflammatory,* have not the effect of causing an increase in the fibrine of the blood as genuine inflammations have. The same is the case with the lesions found in the intestines of those who die from typhus fever; for these pretended inflammatory ulcerations have certainly not the effect on the blood alluded to."

Some continued fevers are attributable to an over-richness of the blood. The synocha, which usually lasts for a few days, is of this character. The term "over-richness of the blood" is certainly one that is vague and even incorrect, if it is meant to imply a superabundance of its fibrine; for in truth there is no increase of this element, but only of the red globules. When *Pinel* published his Nosographie, no reference was made by him to the part which the fluids of the body play in the production of diseases; he placed the seat of what he called "angeiotenic fever" in the blood-vessels themselves;* by his successors it was transferred to the stomach and bowels.

Some authors have given it the appellation of "fever from plethora," or "hy-

---

* Some authors have treated it under the appellation of *angeio-carditis*, and *Tomassini* has denominated it *angeioitis*. Such terms cannot be too soon banished from medical language, as they convey an utterly erroneous view of the disease.

peremic fever"—thus substituting the idea of a humoral origin instead of one that has a reference to any affection of the solids.

In the early stage of other pyrexiæ, such as typhus, measles, scarlet fever, &c. we not unfrequently observe such an inflammatory fever, in which there is a superabundance of the red globules in the blood—a state of the circulating fluid, be it remembered, which does not exist in the genuine phlegmasiæ.

But it is to be borne in mind that there is not any uniform or constant change in the proportion of the red globules in genuine continued fever: it may co-exist with an excessive, or with a normal, or with a defective proportion of this constituent of the blood. We have already said that the proportion of the fibrine is seldom or never increased in the uncomplicated pyrexiæ: frequently it is diminished; and the gravity of the fever is often proportionate to the amount of diminution.* How different from the phenomena presented by the blood in genuine phlegmasiæ, even when they have continued for a considerable length of time, and in spite of blood-letting, low diet and other depletory remedies! "*The organism,*" adds M. *Andral,* "*in the typhoid state exhibits conditions the very opposite to those which characterise inflammation.*"

The congestions of internal viscera, which are so apt to take place during the progress of certain continued fevers, are very generally connected with—we do not say dependent upon—a change in the relative proportions of the fibrine and red globules of the blood, the former element being very generally in a deficient ratio. The same remark is applicable to the occurrence of hæmorrhages during the course of fevers. These two phenomena—congestion and hæmorrhage—are seldom or never observed in the genuine phlegmasiæ.

It is surely quite unnecessary to adduce further proofs of the essential difference between this class of diseases and the pyrexiæ or the genuine fevers.

In conclusion, let us observe that, while we thus insist upon the importance of attending to the various alterations in the fluids which either induce, or are induced by, these as well as various others of the long catalogue of fleshly ills which man is heir to, we by no means wish to undervalue the importance of the pathological study of the solids.

All that we aim at is to impress upon our readers the necessity of a careful examination of both. *Bichat* has strongly stated this truth when he said that, "every exclusive theory of solidism and of humorism is a real pathological absurdity (un veritable contresens pathologique)."

---

### M. Double on the Study of the Greek and Roman Medical Authors.

At the *séance* of the Royal Academy of Medicine, on the 11th December last, M. *Fred. Dubois* read a learned disquisition on the life, writings and doctrines of *Galen.* The interest, with which this paper was received, has excited criticism

---

* Physicians must be on their guard not to be misled in estimating the relative proportions of the elements of the blood by merely noting the bulk of the coagulum, and its relative size to the quantity of the serum. We not unfrequently observe an unusually large clot in the blood drawn from a patient affected with typhoid or other fevers: but then it is almost invariably looser and less consistent than in health. The very deficiency of the normal proportion of its fibrine is the cause of this phenomenon; the more fibrine that is present, the greater is the disposition of the coagulum to contract firmly and expel all the serosity from its meshes. On the contrary, when it is deficient, a more than usual quantity of the watery portion is retained.

from a variety of quarters, both in and out of the Academy, and has prompted one of its oldest and most distinguished members, M. *Double,* whose voice is too seldom heard in public, to address to the author a letter, as remarkable for the elevation of its sentiments as for the finished courtesy of its style. It is always well to listen to the advice of the *chefs* of the profession, and especially of such a man as M. *Double,* who, without having ever engaged in the stormy disputes of the day, has achieved for himself the very highest reputation, alike for great practical skill and for literary attainments.

He has long occupied the president's chair of the Academy, and was lately offered, we believe, the dignity of a peerage by his sovereign.

After some complimentary expressions to M. *Dubois* for having drawn the notice of the profession to "those studies which," he says, "are altogether to my taste, and have rendered many important services to my long medical career," he proceeds:—

"It is in an especial manner the attentive study of the ancient authors that the present generation is deficient in. On every subject which appertains to the general interests of life, the enlightened man will find much in their writings to interest and instruct him. With your tone of mind so eminently critical, and I use this word in its most agreeable acceptation, with your ardent assiduity, and with your love of science, you will open up the classical works of antiquity to the generous youths who are advancing to the sanctuary of medicine. You will speak to them longer and with a loftier and more authoritative voice than I can hope to do, and you will teach them not only how much may be gained from the study of the ancients, but also how much may be lost by neglecting this study. We may draw from the moderns our first and our principal instruction, but let us not forget to refresh ourselves occasionally, nay frequently, at the pure fountains of antiquity,

Comme on boit d'un vin vieux qui rajeunit les sens."

M. *Double* is an ardent admirer of the father of medicine.

"Before the time of Hippocrates there seems to have been nothing but some empirical notions of the art, and some bizarre systems of a philosophy calculated rather to seduce from, than to lead on to, truth. A vast number of scattered observations, and a multitude of individual reports, constituted the knowledge of these days; and it was from these chaotic materials that his genius made our science spring forth. We may therefore say, with Theophilus, that Hippocrates has been the Prometheus of medicine.

Availing himself of all the useful observations preserved in the temples and traditions as handed down to him by his predecessors, he set himself to the task of comparing them with the results of his own observation; and from this two-fold source he deduced those truly beautiful sentences, and those pregnant maxims of profound truth, which all future ages have admired in his astonishing work '*De Aere, Aquis et Locis,*' and also in the treatise on epidemic diseases, that rich model of clinical observation illustrated with all the loftiest powers of intellect, as well as in many of those aphorisms which, by their matter and their style, are of an almost prophetic excellence."

After alluding to the valuable observations of the Coan sage on different points in physiology, M. *Double* dwells with great emphasis on the importance of many of his precepts of general pathology.

"His genius anticipating, and, as it were, improvising the results of experience, has unfolded the two-fold doctrine of the medical constitutions of the seasons —*the defective study of which Science has had so much cause to regret in the present day*—and of the agency of epidemic influences, illustrated by a numerous collection of individual observations: an important groupe of facts, the like of which is not to be found in the writings of any physician during the next 600 or 800 years.

Hippocrates has beautifully shewn that the enucleation, so to speak, of a disease is completely obtained only when the physician has succeeded in discovering its seat, nature and individuality. When this is once accomplished, the true indications of treatment and the most appropriate means to effect this object are readily appreciated by the intelligent physician. These views led the founder of medicine to the distinction of the natural methods of treatment. By studying with great assiduity and skill all the active powers of Nature, or in other words the aggregate of the living functions, in the cure as well as in the production of diseases, he pushed very far our knowledge of the natural methods, and thus prepared the elements of the solution of that vast and important question, viz: to determine in what cases the cure is an effect or product of art, and in what it is a necessary act of nature itself."

Certainly one of the most valuable parts of the Hippocratic writings is that devoted to the exposition of the influence of seasons, climates, age, habits, sex, &c. on the human constitution, and of the necessity of watching and consulting such influences in our management of diseases.

" The old doctrine of temperaments," says M. *Double,* " in my opinion every day more and more rich in clinical applications, and which the experience of ages may modify, extend and improve, but will never overthrow, and the doctrine of diatheses and cachexiæ, a natural deduction from the preceding one, are entirely attributable to Hippocrates. Both are still standing with all their solid instructions and useful applications, in spite of the numerous attacks which they have had at different times to sustain—like those old ruins of Greece and Rome, which, though worn by time and damaged by the hand of man, are still daily visited by the most distinguished artists as models for their guidance and imitation."

M. *Double* gives a rapid and animated picture of the great merits of *Galen* as an anatomist, physiologist and practical physician. So admirable indeed are many of his descriptions of the various organs of the body, not only in man but also in the lower animals, that *Cuvier* confessed that he was utterly surprized and astonished on the first perusal of his writings, and, in his eloquent eulogium on the physician of Pergamus, went so far as to prefer him even to his great model and master, Hippocrates.

Among other remarkable points we may mention that *Galen* was aware that the size of the temporal muscles in animals is strictly proportionate to the hardness and tenacity of the food on which they live; that the number, the form, and the arrangement of the teeth are in a sort of relation or harmonny with the other organs of nutrition and with the *ensemble* of the animal frame; that the heart is double in warm-blooded, and single in cold-blooded animals; that the brain is alternately raised and depressed, and that these movements are dependent on the respiration. His descriptions of the muscles, nerves and vessels is often wonderfully exact. He has started the question, so keenly discussed of late years, as to the distinction of the sensory and motory nerves; he knew that the arteries contained blood and proved this by actual experiment, and also that the veins and arteries communicated with each other—and yet, strange to say, he did not anticipate the circulation of the blood. Who has not read or heard of the beautiful and eloquent description of the hand and foot of man by Galen? of the hand especially, that organ placed in advance of all the other parts of the body as a defence and safeguard, that potent aid of genius, that minister of tenderness and cruelty, that instrument of peace and of war, that agent of creation and of destruction! In a transport of admiration at contemplating these marvels of an Almighty power, he closes his description with these words: " These are the hymns which I have composed in honour of the great Creator of the Universe."

Galen had deeply studied the writings of Hippocrates; he copied, translated, and annotated them. The work of the former, *de locis in homine,* may be viewed as the preface of the beautiful treatise *de locis affectis* of the latter; and in some editions of their works published together, this arrangement is followed, the one being placed immediately before the other.

"I have recently," says M. *Double*, "re-read this treatise of *Galen's* with studious attention; but I must confess that I have not been able to discover in it that spirit or character which you (M. *Dubois*) attribute to the author. You make Galen a *localiser;* but this great statue of antiquity, it seems to me, ill accords with the puny proportions you have given him. Every one acquainted with his writings has agreed in admiring the justness of the comparison which the physician of Pergamus has drawn between the animal frame and the forge of Vulcan, in which, according to the Homeric fiction, all the instruments, instinct with a Divine power, spontaneously moved in the order and with the force required for the work to be done. In this beautiful passage I do not find either the spirit or the language of the localising philosophy of the present day. Similar parts, similar diseases, similar medicaments; the disarrangement in the mutual proportion of the four elements of the body, and the excess of one or of two over the others; alterations in the number, the figure, or the form, the quantity and the situation of the different parts; these are the bases of the doctrine inculcated in the treatise *de locis affectis*—a doctrine which, by the subtle divisions and the speculative opinions it has sought to establish, is aptly criticised in these words of *Boerhaave:* 'multum profuit, multum nocuit.'"

M. *Double* draws an ingenious comparison between Hippocrates and Galen, awarding the palm of superior merit to the former. *Cuvier* had been led, from his insufficient knowledge of the strictly medical writings of the two sages, to adopt an opposite opinion; but the grounds for his preference are obviously attributable to his having almost exclusively studied the anatomical descriptions of the physician of Pergamus; and every one must acknowledge that, on this score, the judgment of *Cuvier* is quite correct. But let it be remembered that six hundred years had elapsed between the death of Hippocrates and the birth of Galen, and that, during this long interval, the human mind had been continually advancing; for centuries are to mankind in general what years are to the intellect of each individual.

"You have said, with truth, that in the writings of Hippocrates we meet with the doctrines of irritation, dogmatism, and empiricism; but it is precisely because we find the germs of all these systems, that none of them can be said to be characteristic or predominant. Faithful to rigorous observation and to reasoning still more severe, Hippocrates has nowhere given his name to any exclusive doctrine. On the contrary, who has not heard of Galenism and its consequences? As long as this system ruled in the schools, and in the practice of medicine, science remained stationary or even retrograded, whereas, when guided by the influence of the Hippocratic doctrines, it has uniformly advanced. The genius of the one sage was calm, severe, and contemplative; that of the other was subtle, brilliant, and discursive."

---

### ON SOME OF THE MOST IMPORTANT DISCOVERIES OF GALEN.

M. *Dubois*, in his reply to the letter of M. *Double*, applies himself chiefly to shew with what truth Galen may be termed a *localisateur*—a term to which his correspondent had objected—by quotations from his works on a variety of subjects. He first alludes to the opinions of his author on the phenomena of the nervous system in health and in disease. . . . . . .

"Examine the most recent treatises on physiology, and observe what is there stated as to the brain being the organ of the intellectual manifestations; then consult the works of Galen, and say, if on this point all succeeding physiologists do not seem to have been eternally condemned to the labour of Penelope!

When you have perused the enormous volume which *Gall* devoted to this very subject, you will probably be tempted to ask, before proceeding further, if some new Archigenes has not appeared to call in question all the facts recorded by him. At the same time do not forget to observe that in a Dictionary, published only the other day, we are told that these facts have been demonstrated only within the last few years. . . . . .

Archigenes himself, although professing a contrary opinion, shewed by his practice that he had adopted Galen's view of the question. In the treatment of all mental diseases, he directed his remedies to relieve the brain—a contradiction which his opponent has tauntingly exposed in these words:—'But oh, most illustrious Archigenes! you are surely not right in addressing yourself to the head, if, in these cases, it is the heart that is affected.'"

M. *Dubois* shews that many other supposed discoveries of modern times were well known to the ancients. Praxagoras and Philotinus had suggested that the encephalon is only an expansion of the spinal-marrow, and that the cranium is only an immense unfolded vertebra; an idea which Galen exposed as an ignorant absurdity.

Erasistratus had maintained that the number and size of the cerebral convolution are *en rapport* with the perfection and extent of his intellectual powers: Galen objected, that if this idea were correct, the brain of the ass should certainly have no convolutions at all;—a poor objection indeed, says M. *Dubois*; for the ass is by no means so stupid an animal; and Homer has not hesitated to compare one of his heroes, Ajax, to it, in consequence of his invincible tenacity and independence of character!

Galen, however, has shewn much more discretion in discussing these subjects than many of the modern writers; for, on one occasion, he stops himself by saying that such discussions lead to questions too lofty and mysterious, but adds, with a natural grace, that it is difficult to avoid treating of the soul when engaged with the organ which is the seat of it. What a fine rebuke to the materialist of the present day is contained in the following passage:—

"If the principle of intelligence be a simple result of the structure of the brain, it would be essentially injured by any change in its texture; but if it be placed there, as we are in our dwellings, it does not necessarily follow that it should be injured by such physical changes. As, however, philosophers are not agreed on this question, and as they are unable to tell us whether the mind is an accident or result of matter, or whether it be merely associated with it, we should be satisfied with the simple announcement of the fact, that lesions of the encephalon generally induce disturbances of the mental powers."

. . . . But to return to the subject of localisation, we should remember that Hippocrates himself has distinctly stated that, when one lateral half of the brain is injured or diseased, the affection is manifested on the opposite side. Aretæus is more explicit in his description of this pathological fact, and gives an anatomical explication of it. Strange to say! this important truth became almost entirely forgotten; for we find that *Pinel* has not even alluded to it in his account of apoplexy, although in his report of *Daubenton*'s last illness, he expressly mentions that the left side of his body was paralysed, and that nearly two ounces of blood were found on dissection in the right ventricle of the brain.

Now let us ask ourselves if modern discoveries have added anything of value to the proposition enounced by Hippocrates and Aretæus. Can we, with any degree of certainty predict the seat of the lesion, or say whether the brain itself or its meninges are affected in any particular case? All that we can do is to make some conjectures; and *Galen* had long ago shewed how unsatisfactory and futile these usually are. 'We need not,' says he, 'trouble ourselves about trying to discover whether it is the brain or its coverings that are most affected; since, in either case, we know to what side of the head we should apply our remedies.'

So much for the encephalon; let us now turn our attention to the spinal-marrow. It has been formally announced in our days, that the symptoms of a lesion of this organ are manifested on the affected side. What says *Galen* on this subject? "You have seen that when our incisions, made transversely on the spinal marrow, extend to only one-half of its thickness, the parts situated below the place of the incision were immediately paralysed, but only upon the side of the section; on the right, if the right half was cut, and *vice versâ*." . . . . . .

*Galen* was also well aware of the different effects of external violence or disease on the spinal-marrow in different parts of its extent, and that the fatal consequences of the cervical portion being injured are attributable to the interruption of the breathing thereby induced.

It is really astonishing to observe the singular accuracy of the old physician's knowledge as to the functions of the nervous system, and the effects of injury or disease upon them. He had even, in some measure, foreseen that beautiful discovery of modern times, the distinction between the nerves of sensation and those of motion. He confessed that he was unable to solve the question: "grand problem!" he exclaimed, "question full of difficulties and doubts! which Herophilus and Eudemas have left to posterity for solution!" And how many centuries passed before posterity ever applied themselves to solve the question, which had been *formulé* in so plain and distinct a manner by *Galen*.

If our space allowed, we might also allude to his clear-sighted views on the localisation of the intellectual powers in the anterior part of the cerebrum, while at the same time he avoided most of the errors into which the hasty curiosity of many modern physiologists has fallen, in their attempts to define the seat of each mental faculty. While he was aware that one faculty, as the memory for example, might be specially affected, he confessed his utter inability to arrive at any accuracy in his attempts to establish a local diagnosis of the seat of individual faculties. Whoever will carefully peruse the writings of *Galen* cannot fail to be struck with the singular sagacity of his views on the functions of the nervous system. Had they been better known by physiologists, and had the example of cautious deduction which he has set, been more generally followed, more progress might have been made in revealing some of its mysteries than has been by the mode adopted of late years.

M. *Dubois* closes his remarks with some allusions to that passage in *Galen*'s life, which, if true, is but little creditable to his feelings of humanity and justice —his cowardly desertion of his post of duty when the plague broke out in Rome.

He has not, however, been without finding excusers of his alleged conduct on this occasion. M. *Double* benevolently doubted the truth of the charge; but M. *Dubois* seems satisfied that there are ample grounds for it, and attributes his unworthy behaviour to the general demoralisation then prevalent. "The true motive," says he, "is, that in Roman society at that period the *moral sense* was nearly quite lost, more especially among the learned and affluent; for the regeneration, which Christianity was preparing, shewed itself as yet only among the lowest classes of society."

Galen was about thirty-four years of age when he arrived at Rome, where he soon acquired so high a reputation that his practice increased even beyond his wishes, *cum ægrorum curatio felicius quam optassem succederet*. He was probably rather a boastful loquacious gentleman, for he tells us himself that his *confreres*, who were probably jealous of his rising success, called him a *logiatre! moxque logiatron exclamarent*. He therefore resolved that from this time he should keep a rein upon his tongue, and never say more in consultation than what was absolutely necessary: *ut invidam istorum linguam declinarem, non, apud illos quos curabam, plus quam necesse erat profabar;* and he adds, *ne me plusphilosophi ac*

*medici delusorem et vatem appellent.* He tells a capital anecdote of his "savoir faire" in imposing on the credulity of some of his cotemporaries, who from his account seem to have rivalled in skill many of the *dispensing doctors* in the present day. We should suppose that he was a sly humourous rogue in his way, laughing in his sleeve at the ignorance of some of his confreres, and all the while keeping a sharp look-out at the main chance.

We extract the following report from his work De Locis Affect. libr. v. c. viii: it is really very amusing.

---

### Galen's Report of a Case of Hepatitis.

"Soon after my arrival in Rome, Glaucon, the philosopher, took a great fancy to me in consequence of my reputed skill in the diagnosis of diseases. Meeting me one day in his walk, he stopped to shake hands, and thus accosted me: You are the very person I want; I have this moment left one of my friends, a medical man himself whom you may have seen with me the other day, very ill; suppose we go and call upon him.—Where does he live, and what's the matter with him? I said.—Glaucon, who is of a most frank disposition, replied; I have heard from Gorgias and Appelles that you have on more occasions than one given such an accurate diagnosis, that it seemed more like the result of divine inspiration than of human knowledge; and I am glad of this opportunity of putting to the test this talent of yours, that I may satisfy myself whether you really have this marvellous faculty of presaging the most obscure questions.

By this time we had reached the door of his friend's house, so that I had not even a moment to tell him, as I have so often done to you, that, if in some cases we have one or more of those symptoms which leave no doubt as to the nature and issue of an existing disease, in other cases they are so far from being satisfactory or unequivocal that we require a second or even a third examination of the patient before we can with any confidence pronounce our opinion.

As we entered the house, I observed in the first vestibule that a servant was carrying from the invalid's room a vessel containing a serous fluid, which appeared to be slightly sanguinolent and not unlike the washings of fresh meat.

This at once satisfied me that there was some affection of the liver;* but I made no sign in any way of what I had observed, and we at once proceeded to the patient's room.

The first thing I did was to feel his pulse, in order to determine whether the affection was of an inflammatory nature or not. As the patient was a medical man himself, he remarked that his pulse might have increased in frequency by the fatigue and exertion of getting up to stool; but I had already assured myself that his complaint was strictly inflammatory.

I observed at the same time on the selle of his window a pot which contained what seemed to be hyssop mixed with honey and water; nothing more was required to shew me that our gentleman thought that he was labouring under a pleurisy.

This idea was probably suggested by the sharp pain which he felt under the false ribs—a symptom however which is very common in inflammation of the liver also—by the cough, and his short hurried breathing.

Having thus learned all that was necessary, and profiting of the opportunity

---

* The ancients were in the habit of regarding the serous evacuations from the bowels at the commencement of dysentery and other intestinal affections as proceeding from the liver, and hence they gave the name of *hepatorrhea* to this symptom.

which luck put in my way of giving Glaucon a high idea of my skill, I placed my hand over the false ribs of the right side, and told the patient that *there* was the seat of the disease: he admitted that it was. Glaucon, who supposed that it was only from feeling the pulse that I had made the discovery, was at once delighted and surprised.

To increase his astonishment, I said to the patient; 'besides the pain in the right side, you are distressed every now and then with a troublesome dry cough, with little or no expectoration.'

While I was yet talking, he was seized with just such a fit of coughing as I had described. Glaucon could no longer conceal his surprise, and forthwith began to praise me to the skies.

Wait a little, I said to him; do not suppose that this is all that my art enables me to do. Then addressing myself to the patient, I continued,—do you not find the pain to be increased, and feel a sense of weight in the right hypochondrium, whenever you take in a deep inspiration? he at once replied that it was so, and seemed to be almost as much astonished at my prediction as Glaucon himself.

Seeing that fortune continued to smile on every thing that was done, it occurred to me to say something about the shoulder; for I was well aware that in most diseases of the liver there is a feeling of dragging down of the right shoulder. I had no sooner made the allusion than the patient at once confessed that it was just as I had predicted.

I will add only one other word more, I said; I will tell you what you have supposed was your disease. Glaucon exclaimed that he should not now be surprised to hear me divine even that too. The patient, astonished at my confidence, looked at me most steadfastly, awaiting with anxiety what I was about to say.

I told him that he believed that he was labouring under a pleurisy; he immediately answered that it was really so, at the same time expressing his profound veneration for my skill. His medical attendant also had formed the same opinion; for he had just ordered an oily fomentation to the side, such as is commonly used in this disease.

From this period, Glaucon formed the very highest opinion of my professional abilities, although hitherto he had always spoken slightingly of medicine and of physicians; but it must be confessed that he had probably never come in contact with any one of consummate skill.

I have detailed these particulars, adds *Galen*, in order that, if fortune throws in your way a favourable opportunity, you may know how to turn it to the best account; for it not unfrequently happens that we are placed in situations by which with good tact we may reach celebrity, although the greater number of men from ignorance cannot avail themselves of the chance."

(A very good commentary on the well known lines—

There is a tide in the affairs of men,
Which, taken at the flood, leads on to fortune;
Omitted, all the voyage of their life
Is bound in shallows and in miseries.

Few men feel the truth of the maxim with greater force than many of our own profession.)

---

### BIOGRAPHICAL NOTICE OF RICHERAND; HIS DISPUTES WITH DUPUYTREN, &c.

*Richerand* was born in 1779, and, while yet a young man, went to Paris with the view of establishing himself there as a surgeon. Gifted by nature with quick vivacious talents, he early distinguished himself as a ready and graceful writer.

In the 22nd year of his age he published his well-known Elements of Physiology—a work which has passed through very numerous editions, and has been translated into almost every European language. It will long remain a favourite with the medical student, from the beauty of its style and the liveliness of its descriptions; indeed, there is perhaps no professional book which has been so extensively known to general readers as this model of elementary works.

Every celebrated man has, it is said, his peculiar fatality; that of *Richerand* was to find in his youth such a rival as *Bichat,* and in his manhood a competitor like *Dupuytren.* He was surpassed by both, but without being *effaced.* The sun of *Bichat,* which rose with such brilliancy of promise, soon set; and *Richerand* then reigned nearly alone in the path which he had opened up to himself. He had dedicated his work to *Fourcroy,* who at that time occupied a high post in public instruction, and he was not long in receiving the reward of his well-selected homage. He was appointed surgeon first to St. Louis Hospital, and afterwards to the Guard of Paris; and in 1807 he obtained the chair of surgical pathology in the Ecole de Paris.

He devoted himself with great energy to the labours of his profession, and at length acquired a high reputation as a bold operator. This was greatly increased after the occurrence of the memorable case of cancer of the breast, in the removal of which he cut away portions of several of the ribs. The case was altogether a very extraordinary one. The patient was a surgeon himself, and the disease had made so much progress that the subjacent ribs had become affected. Encouraged by the able counsels of his fellow-professor *Dupuytren,* M. *Richerand* undertook the formidable operation of excising the whole of the morbid mass. "By a quadrilateral opening which formed a sort of window in front of the heart," this organ was quite exposed, and was seen and touched by several persons. (Histoire d'une resection des Côtes et de la Pleure, lue à l'Academie Royale des Sciences, 27 Avril, 1818). Although the patient died subsequently from the return of the disease, this "belle operation" was not the less creditable to the skill and daring of him who performed it; and his fame was widely diffused in consequence. But unfortunately for him, he was not able to maintain the high position to which he had now been raised in the eyes of the public. He did not combine the calm penetrating glance of the philosophical surgeon with the boldness and sang-froid of the skilful operator. "No one," said his lively compatriot *Brillat Savarin,* "could comfort better, or had a gentler hand or a more rapid use of the knife:"—true; but such qualities, although they indicate talent, can lay no claim to genius. Now, this was the case with *Richerand.* And then beside him, and almost always under his very eyes, there was rising up a rival who knew how to combine mental powers, that were unquestionably superior to those of *Richerand,* with an insatiable ambition, and with a daringness, a self-confidence, and at the same time a pliability of character that had rarely been equalled.

This man had moreover a great advantage over his rival,—a masterly talent of speech. *Richerand,* although a graceful and eloquent writer, could never express himself in public well. He seemed to be confused in his thoughts, and his language was abrupt and inelegant. He had none of that *flumen orationis,* that smooth strain of animated words which captivates an audience and gives confidence to the speaker. His accent and pronunciation were moreover bad, and his gestures utterly graceless.

All this was much against his success as a professor, and the very consciousness of his defects, which were rendered still more conspicuous by the foil of *Dupuytren's* admirable talents as a public speaker, seemed to annoy him exceedingly, and was probably the main reason of his deep-sown envy against his celebrated colleague.

The publication of the *Nosographie Chirurgicale* added greatly to his reputation, and deservedly so; but, like all his former writings, it was chiefly conspicuous for the beauty of its style, and a certain felicitous explanation of whatever subject was treated of. In the course of a few years, however, its success began to wane before the increasing brilliancy of *Dupuytren's* lectures—lectures which, it is well-known, attracted students from every part, not only of France but of Europe, to listen to the words of the Napoleon of surgery. *Richerand's* class-room on the other hand was never well attended. *Desgenettes,* ever ready to launch a biting jest when an occasion offered, replied to a gentleman—who asked, "what is *Richerand* doing? is he writing?"—No, our colleague "professe un cours public que le public evite."

When the Academy of Medicine was instituted, *Richerand,* whose elegance of style was well-known, was appointed secretary of the surgical section. Impelled by the desire to rule, a feeling which almost always attends men of some talent, he wished to have an ascendancy in the meetings, and to imitate the example of *Louis,* the illustrious secretary of the old Academy of Surgery. But here again he failed in his object. The frequent opposition which he encountered, and the satirical allusions to the new *demi-Louis,* soon taught him that his pretensions could not be maintained; and accordingly he resigned his situation. The presence of his rival too was a source of continual vexation; feeling, as he must have done, how he was overshadowed by the superior talents of *Dupuytren,* whose disdainful hauteur, and cold contemptuous manner were always blended with an air of affected moderation. The virulent attacks and bitter personal recriminations, in which both too frequently indulged at the meetings of the Academy, are still remembered with regret. On one occasion, a member of the learned assembly exclaimed with most malicious point, "These gentlemen tell *such truths* of each other, that those who hear them take them for calumnies." The quarrel ceased, but not the mutual hatred which had given rise to it. *Richerand,* in a public report communicated to the Academy, and which he subsequently published in an extended form, under the title of the "Recent progress of Surgery," attacked his enemy under the guise of *Simon Pimpernelle,* who is described in an old work as *Parisiensis, vir disertissimus, consultor famosus, societatis barbitonsorum chirurgorum quater præfectus;* and on a subsequent occasion he designated him as "a man with a heart of ice, and a brain circled with bronze, who lies as other people breathe."

It is a curious circumstance that *Richeraud* was, after the death of his great rival, charged with the article *Dupuytren* for the Biographie Universelle. Although a reconciliation had taken place during the last illness of the latter, and though the writer seemed to have felt the justice of the saying, *nil nisi bonum de mortuis,* he was not able to conceal the old spirit of animosity which had so long existed between them: *manet ultâ mente repostum.* After bestowing a few eulogies, as pale as insignificant, he sums up the character of *Dupuytren* by telling us that his genius "ne consista qu' *à faire autrement.*"

The article was ill-received by the public, and gave rise to the witty remark of Dr. L., "*Zoilus genuit Mævium, Mævius autem genuit Richerandum.*"*

He afterwards, we are told, commenced a history of surgery; and it is to be regretted that he did not carry out his design to completion. Instead of this, he entered the arena of political partisanship, and published, in 1837, a work entitled, "Of Population in its relations with the nature of Governments." It was

* As a specimen of the article we may quote the following passage: "An eloquent, ready, and ingenious professor, gifted with an indefatigable activity. To have his name repeated with the epithets of the great, the eminent, the first surgeon of the Hôtel Dieu, seemed to be the main object, as it was the chief delight, of his life. It consoled him for his domestic misfortunes, the publicity of which was not without its charms to him." (*Dupuytren,* we believe, was unfortunate in his matrimonial alliance; his wife eloped with a foreigner.—*Rev.*)

a hasty production, and had but little success: in truth it was not much more than a vehement invective against the nineteenth century. His views seem to be those of ultra-toryism. He is friendly to an almost absolute despotism, and regards liberty and equality as sublime political *niaiseries*. *Montesquieu*, according to him, is only a cautious Gascon; and *O'Connell* is a revolutionary Thersites. In politics, as in surgery, he invokes the use of fire and steel for the cure of social as of corporeal disorders. A devoted admirer of the silence and order which prevailed under the rule of *Napoleon*, he seems to have entirely overlooked the disastrous close of the mighty emperor's life. He disliked every novelty, and the social movement, that is going on in the present day, was viewed by him only as the baneful work of disorganisation. He was ever afraid, like the Abbé *Sieyes*, of seeing "l'enfer" of the republic reinstated in power.

Although of a strong and vigorous constitution, *Richerand* did not live to be an old man. The serene influence of easy circumstances, and of a life apparently calm and fortunate, did not contribute to lengthen his days. He died after a short illness in January 1840. He had expressed his desire that no *eloge* should be pronounced over his tomb; well aware, no doubt, of what little value such posthumous glorifications are in the present age of scepticism and indifference.

It is useful to every one to muse occasionally on the characters of the leading men in our profession; especially after they have been withdrawn from our eyes, and when all partiality and prejudice have ceased to influence our judgments. The lives of such men as *Dupuytren* and *Richerand* may suggest many a useful hint to the medical practitioner. Both were gifted with great talents; but both alas! were destitute of that well-adjusted poise of mind, and that guidance of the higher moral feelings, without which, although intellectual and professional eminence may be attained, the real prosperity of personal happiness and of cotemporary respect is not to be won.

Let it be ever borne in mind that no person can continue to give way to the impulse of any one extreme and energetic feeling, without compromising his own comfort and welfare. *Dupuytren* and *Richerand* suffered most bitterly from this fatal mistake. And what has been the result?—not only were they unhappy in themselves, but they also failed in doing for science what science had a right to expect from them. Both of them died in the strength of their age, worn out less by the decay of their bodily powers, than by the corrosion of mental anxiety and disappointment. In the latter years of *Dupuytren's* life, notwithstanding his ever-increasing celebrity and worldly success, the care-worn expression of his features, and the cold mechanical smile that often played upon his lips, indicated too clearly the inward distress and deep-rooted melancholy which was preying upon his soul. The germ of death was already sown. His reputation with posterity will not be at all commensurate with the great fame which he had in his day. *Richerand*, on the other hand, vexed at the acknowledged superiority of his rival, wasted his talents in angry contentions, and at length gradually retired from the field in which, had he been satisfied with the second command, he might have won most honourable laurels. The manhood of his professional career by no means fulfilled the bright promise of his early days; not that his capacities and attainments had at any time been over-estimated, but simply because they were not directed in a right course, nor matured under the fostering influence of a peaceful and contented spirit.—*Gazette Medicale.*

---

### Memory of M. Sanson.

We observe by a notice in the Gazette Medicale that this distinguished surgeon and truly honourable man recently died in Paris. Although much respected by every one, his pecuniary affairs seem to have been almost always embarrassed; for we are told that, "after having constantly lived in the most respectable poverty, he left scarcely sufficient means to pay the expenses of his funeral." We are glad to observe that a committee of his professional brethren was immediately formed for the purpose of raising a subscription to erect a *modest* monument over his remains, in token of their admiration of his talents and respect for his moral character. The names of *Chomel, Royer-Collard, Rayer, Cruveilhier, Dubois,* &c. are put down for 100 francs each. M. *Sanson* had been long in the army, and for some years past was one of the surgeons of the Hôtel Dieu.

---

### Circumcision as practised at Constantine.

Dr. *Bonnafont,* one of the physicians of the French army in Algeria, was on several occasions witness to this operation as performed by a native surgeon or *tebib.* The time at which it is usually practised is about seven or eight years of age. There is generally a festive meeting of the friends of the parents assembled on the occasion; but they all take their leave before the ceremonial rite is commenced. On one occasion, at which Dr. B. was present, upwards of thirty friends breakfasted; they then washed themselves and were sprinkled over with rose water, and after praying they all departed. Seven large candles, fixed in a rod of wood, and ornamented with ribbons or coloured paper in a fantastic manner, were then placed at the side of two cushions laid on a low seat; and at the other side there was a vessel holding wood-ashes, and another in which some paper was burning. The father sat down "a cheval" on the cushions, and held his son before him in nearly the same position as we employ in the operation of lithotomy. The *tebib* seated in front first forces back the prepuce so as to uncover the gland, which he dusts over with the ashes of the burnt paper. This forcible retraction of the prepuce sometimes gives much pain. The *tebib* then pulls it forward again, squeezing back the glans as far as possible, and working the prepuce freely between the fingers. Holding it firmly between the nail of his thumb and the forefinger of the left hand, he then snips it across with strong scissors. This is so quickly cut that the child has no time to cry out before it is all over. The wound and the whole of the penis is then dusted well over with the ashes of the burnt wood and paper; nothing else is applied. A fresh application of the ashes is made every morning. There is thus gradually formed an impermeable crust, which comes away like the finger of a glove, when the cicatrisation is complete—this being usually the case about the 25th or 30th day after the operation. During all this time the child is an object of especial regard to his family and his friends, who are every now and then visiting him, and shewing him all marks of kindness.—*Annales de la Chirurgie.*

---

### Transmission of Glanders from one Human Subject to another.

M. *Berard,* surgeon of the Necker Hospital in Paris, has communicated to the Royal Academy an interesting case which, in his opinion, clearly establishes this important but melancholy fact.

One of his pupils had the charge of dressing an ostler who was admitted with the symptoms of chronic farcy, and subsequently became affected with acute glanders; he eventually died. The dissection was performed with great care by M. *Rocher* (the pupil), who, while the sawing of the cranium was performed, kept his hands on the face, which had been affected with a gangrenous eruption. A short time before the death of the patient, he (M. *Rocher*) had been affected with colicky pains and slight diarrhœa; but it was not till the night after the dissection that he became seriously ill. He awoke with a shivering fit, which was soon followed with the usual symptoms of fever. The pains in the limbs were severe, and on the third day fixed themselves in the left thigh, the right shoulder, and the right side of the chest. On the fifth day, M. *Berard* detected in these parts puffy swellings, which had many of the characters of farcy tumours; his prognosis was therefore very unfavourable. The swelling in the right shoulder disappeared; but that in the left thigh increased, and was laid open with the knife: a quantity of pus mixed with blood was discharged.* Another tumour, preceded by most severe pains, made its appearance over the right ankle, and proceeded on to suppuration. On the 14th day after the invasion of the disease, the skin of the nose became red and painful; on the following day, the redness had extended to the cheeks, eyelids, and part of the forehead, and here and there several gangrenous phlyctenæ were observed. Next day numerous pustules appeared on different parts of the body, and a copious discharge issued from the nostrils. The patient died in the course of the night, sixteen days from the commencement of his illness.

The horse that had been inoculated died upon the same day, after having exhibited the symptoms of acute glanders; the nasal fossæ presented the usual morbid appearances of the genuine disease.

M. *Berard* says that there had not been any wound or puncture of the integuments in the case of his regretted pupil, and that he had always taken the precaution of washing his hands after dressing his patient. He is therefore of opinion that the disease was communicated by miasmatic contagion in the same way as small-pox and other diseases are known to be propagated. The report of the case has been sent to the surgical committee to investigate its particulars with attention.

M. *Bouillaud* stated that he had recently at La Charité hospital a case, the nature of which he had not discovered till within a day or two of the patient's death. He was a youth, who had been admitted into M. *Velpeau's* ward in consequence of a painful swelling over the sternum. There was slight œzena, and incipient erysipelas of the face; the breath was very fætid, the lips dry, and the tongue was covered with dark spots. He was bled, and the blood was buffy. On the following day he became delirious, and numerous pustules had made their appearance on different parts of the body, but chiefly on the face. It was the occurrence of these pustules that first suggested to M. *Bouillaud* the idea of the glanderous nature of the disease. The patient died; and on dissection several purulent deposits were found in the muscular tissue; some of the pustules had proceeded on to ulceration. In the nasal fossæ there were several minute grey ulcers, varying in size and depth; one large one was situated at the top of the pharynx.

The case had been viewed from the first as one of bad typhus fever; but there can be no doubt now that it was really one of acute glanders. The youth, it was afterwards discovered, had been in the habit of dressing two omnibus horses, which were labouring under the disease.

At the same meeting of the Academy a case was related by one of the pupils of M. *Rayer* at La Charité, in which one of the grooms of the great veterinary school at Alfert died of acute glanders supervening upon the chronic form, which had continued for a number of months, and had unfortunately been mistaken for syphilitic disease, in consequence of the palate and throat being chiefly affected. Besides the usual lesions in the nasal fossæ, &c., the palate bones were found eroded in several points, and the entire length of the trachea exhibited traces of

---

* A horse was inoculated with some of this matter by M. *Leblanc*, the well-known veterinary surgeon of Paris.

previous mucous ulceration, which had cicatrized. Such an appearance is not uncommon in horses that have been affected with farcy or with chronic glanders; but it has not been observed in the human subject before the present case.

From the preceding statements it would seem either that glanderous disease in man is becoming of more frequent occurrence, or that many cases, which used to be regarded as instances of syphilitic or other morbid infection, are now found to be owing to the contamination of equine virus.

---

### HINT FOR THE TREATMENT OF DIABETES MELLITUS.

M. *Bouchardat,* in a memoir recently communicated to the Academy, remarked that the matter of perspiration is usually found to have lost its normal acid properties, while on the other hand the secretion from the mucous membrane of the digestive canal, which is alkaline in health, becomes acid. He strongly recommends brisk friction of the surface, with the use of warm flannel clothing, and the internal administration of carbonate of ammonia, to restore the healthy state of the cutaneous and mucous secretions.

*Remark.*—That diabetes mellitus is essentially a humoral disease, or in other words dependent upon or connected with an altered state of the fluids of the body, there cannot be a doubt; and it is therefore highly probable that more light may be thrown on its real nature by the discoveries of organic chemistry than by any other mode of examination. Whether M. *Bouchardat* is correct in stating that the cutaneous secretion changes from acid to alkaline, and the mucous secretion from alkaline to acid, we are not prepared to say: we must await the results of future experiments.

Dr. *Willis,* in his recent work on Urinary Diseases, alludes particularly to the good effects of magnesia in the treatment of many cases of diabetes mellitus, and other practitioners have had occasion to make a similar remark.* Perhaps however this need not surprise us, considering that, whenever there is any disturbance of the digestive functions, there is always a greater or less tendency to acidity of the stomach. No doubt the use of an animal diet must powerfully contribute to obviate this tendency whenever it exists.—*Rev.*

---

### FATAL CASE OF ACUTE PLEURISY AND EFFUSION; WITH PRACTICAL REMARKS ON THIS DISEASE.

A middle-aged man, soon after recovering from a protracted attack of dysentery, was suddenly seized with pain in the left side of the chest, which had been gradually increasing for eight or nine days before he applied for medical relief. The pain at this time was very severe at one point immediately below the mamma; it was much increased by deep inspiration, and by the cough, which was dry and of a convulsive character, returning frequently in fits of five or six minutes' duration. The patient could not lie upon his right side; the easiest position being on the back. Percussion elicited a clear sound from the right side of the chest; but there was a dull resonance over the lower half of the left one. The respiratory murmur however was, we are told, perceptible on both sides; but it is added afterwards, " we thought that we could hear an œgophonic sound over the seat of the pain." (This remark seems to be the result of an *arriere-pensée,*

* Vide the Medico-Chirurgical Review for July, 1839.

*Rev.*) The pulse was rapid and tolerably firm, and the breathing was much hurried.

On this day (7th of Nov.) he was bled to twelve ounces, and twenty leeches were applied over the seat of the pain. The bleeding was repeated in the course of the evening, in consequence of the severity of the cough: the blood on both occasions was covered with a dense buffy coat. On the following two days he was again bled, and on the latter, a blister was applied on the left side. A mixture with syrup of poppies and nitre was also ordered at the same time. The symptoms were somewhat relieved, but only for a short time; for on the 11th we find that the cough is reported as being as troublesome as ever, and the pain still severe: " persistence also of the dullness of the chest and of the absence of the respiration." (This is the first notice of any absence of the respiratory murmur.) The blister was ordered to be kept open; and the patient was advised to inhale the vapour of an infusion of belladonna flowers to relieve the cough, which was still most distressing. The expectoration had hitherto been very scanty, but about this time (14th) it began to be more abundant. The whole of the left side was now dull on percussion, and a bronchial respiratory sound was audible over its entire extent; the pulse and breathing were still very rapid, although the pain was considerably less, and the cough was a good deal abated. The doses of the nitre were considerably increased in the hope of stimulating the absorption of the effused fluid.

The dyspnœa, however, became more and more distressing, and the only position in which the patient could lie was on the left side: the number of the respirations was nearly sixty in the minute, and the pulse was rapid and feeble. The operation of paracentesis was therefore performed without further delay: about eight ounces of a transparent serosity flowed from the canula, and the discharge was then stopped. The patient experienced considerable relief for some hours afterwards; but, the dyspnœa again increasing towards evening, eight ounces more were withdrawn. A quiet night followed, and altogether the patient was easier next day.

On the following days (17th and 18th) eight ounces were again withdrawn each time by the canula, and nearly as much flowed out from the wound by the side of the instrument: a few bubbles of air escaped the last time. On the 19th, the resonance of the affected side on percussion was greater than in health; but no respiratory murmur could be heard on auscultation: bubbles of air continued to ooze from the wound. The canula was therefore taken out. For two or three days a considerable diarrhœa had been present, and tended to exhaust the patient's strength. On the 25th, a mucous rale was audible on the right side, and a slight gurgling and amphoric sound on the left; the air entered and escaped freely through the wound during the acts of coughing especially. On the 28th he died.

*Dissection.*—On making an opening into the left cavity of the chest, a quantity of fetid gas made its escape, and some purulent matter was found within. The pulmonary and costal pleuræ were invested in almost their entire extent with a false membrane. The lung on this side was rather denser than in health; but it readily crepitated on pressure. The right lung was nearly sound, and the heart was quite healthy. The viscera of the other cavities exhibited no unusual appearances.

(*Remarks.*—There are few diseases, the diagnosis and the successful treatment of which require more tact on the part of the physician than pleurisy accompanied with effusion into the cavity of the chest. As long as the symptoms are acute—the pain in the side being very severe and much aggravated by a deep inspiration or by coughing, and the symptoms of synocha being present—there can be no difficulty, as a matter of course, in determining the proper treatment to be adopted, whether we have reason or not to suspect the existence of fluid in the pleuritic cavity. A vigorous antiphlogistic practice must be pursued until the inflammation is subdued. But in the majority of cases the symptoms are much more perplexing, when the effusion has once taken place. The pain has usually abated a great deal; but the breathing is more oppressed, and the general anxiety of the patient is much greater. He cannot lie upon the healthy side; and sometimes the only position in which he can find ease is on his back. In certain cases, even when the effusion may not be very great, the dyspnœa amounts to more or less complete orthopnœa. The cough is usually distressing, recurring at short intervals in fits of great severity, and it generally resists every form of opiate or other narcotic medicines. No sooner has the patient fallen asleep than he is probably awakened with a paroxysm which quite tears him to pieces, and the severity of which leaves him much exhausted for the time. The cough is generally dry, hard and shrill—it is always worse when the head is laid low. There is usually little or no expectoration. This peculiar convulsive sort of cough, the distress in the breathing in the reclining position, the inability to lie on one or on both sides, taken in conjunction with the auscultatory signs—the dullness on percussion of the affected side over a greater or less extent, and either the absence of all respiratory murmur or the substitution for it of a bronchial sound—are the characteristic indications of pleuritic effusion. An unpleasant symptom, and one too very generally present, is an extreme rapidity of the pulse, which is almost always weak and compressible at the same time.

Not a few cases of this dangerous malady are mistaken by medical men for bronchitis, or for phthisical disease of the lungs, and they often go on prescribing for the symptoms for days and weeks, without even suspecting the existence of effusion into the chest. We have the authority of the late Dr. *Hope** that more than one eminent physician of this metropolis has, to his knowledge, mistaken the nature of such cases, so far as even to recommend their patients to try the effect of a residence in a warm climate, erroneously supposing that they were labouring under tuberculous consumption. It is unnecessary to dwell on the treatment of pleuritic effusion, as we fully concur in all respects in the judicious advice given by this lamented physician. The course pursued by Dr. *Lequine* (the narrator of the preceding case) was feeble and most unsatisfactory. He seems not to be aware of the powerful effects of mercury, especially when combined with squills and digitalis, in promoting the absorption of the effused fluid; neither does he appreciate the great value of repeated large blisters to the affected side—and yet these are the most efficient of all remedies. Then again the practice of leaving the canula in the wound made by puncturing the chest, so that the air readily passed in and out of the chest, cannot be too severely reprobated. There may be a few cases, when the disease has been of old standing and the constitutional symptoms are not severe, where this may be hazarded; but, as a general rule, the wound in the chest cannot be too quickly healed. Fortunately however the extensive experience of Dr. *Hope*, and also of Dr. *Stokes* of Dublin, has fully proved that the operation of paracentesis thoracis is very seldom necessary, as the effused fluid may generally be caused to be absorbed by the use of judicious internal remedies.—(*Rev.*)

### Cases of Milky Urine.

In our review of M. *Rayer's* elaborate work on the diseases of the kidneys in our last number, we directed the reader's attention to this singular malady. We

* See his admirable unfinished Paper on this subject in the Medico-Chirurgical Review for last July.

mentioned that it has been observed in several cases to occur in patients, who had laboured under hæmaturia, more especially that form of it which seems to be endemic in the Mauritius, and in some other tropical countries. The following cases, from the work of M. *Chapotin* to which we then alluded, are illustrative of our remarks.

*Case* 1.—A young creole of the Isle of France had been subject in his youth to hæmaturia, which had continued till he was 14 years of age. His health, however, remained good, and did not seem to be affected until his seventeenth year, when he was attacked with sharp pains in the loins, which, after lasting for a few days, were followed by a discharge of urine of a milky appearance. This state of the secretion had continued for about two months, when the patient first consulted me. He was thin and weak, but did not complain of pain anywhere. His appetite was tolerably good; but his digestion seemed to be feeble; and he had always more or less thirst upon him. The bowels were relaxed several times in the course of the day, and after the least exercise the skin perspired copiously.

The urine, which was less in quantity than the drink taken, exhibited on cooling a whitish coagulated mass like the curd of milk, and gave out a peculiar faint smell. From this clot, when squeezed, there flowed out a whitish serosity, which upon analysis afforded a considerable quantity of fibrine. The action of boiling water and of sulphuric acid showed the predominance of albumen; the quantity of gelatine was much less. The urine contained scarcely any acid, and only a very small proportion of its ordinary salts.

M. *Chapotin* recommended a nutritious animal diet and the internal use of steel and bitters, with the application of stimulant liniments to the loins and abdomen. Under this treatment, the patient's general health improved very sensibly; but the state of the urine remained as before. The tincture of cantharides was then given internally: in the course of ten days the dose was increased from six to twenty drops. The beneficial effect of the remedy was soon apparent: first the fibrine, and then the albumen and gelatine disappeared, and the urine gradually acquired a yellowish hue in proportion as these elements diminished and that of the urea increased: its odour, however, remained still faint and unpleasant. In the course of another week or two the secretion appeared to be perfectly normal, and continued so for nearly two years under the use of a tonic diet, the use of cold bathing, and residence in the country. He had then an attack of nephritic colic, in consequence of some imprudencies, and again the urine became of a whitish hue; but this appearance quickly vanished under the use of very simple means.

Dr. C. mentions that he has cured three other cases with the same remedies which he employed in the preceding one.

*Case* 2.—A gentleman, native of the Isle of France, 21 years of age, had been affected with hæmaturia in his childhood; in his 14th year he suffered from occasional nephritic pains, but did not void any gravelly matter at the time. In 1833, after lifting a heavy weight, he passed a great quantity of blood by the urethra. In 1835 he left the Isle of France for Paris. While there, the urine was occasionally thick and sanguineous, with a whitish colour on the surface: these symptoms were always increased by any bodily fatigue, and were usually accompanied with pain in the loins. In 1836 he consulted M. *Rayer*. His urine, when allowed to rest in a closed phial for some hours, was found to exhibit a white layer on the surface. On the following day, the same specimen had separated itself into two distinct nearly equal portions, of which the upper was opaque, of a yellowish-white hue, and without any sensible odour, and the lower was red and opaque, and had deposited two coagula—one of which was reddish-brown, like a clot of blood, and the other was white. The upper portion was found by chemical tests to contain a fatty matter of a beautiful yellow colour, and which left a strong oily mark on paper. (The details of the analytic examination are given at considerable length by our author, to whose work we must refer those who wish to know all the particulars.)

The patient was ordered to be bled, and keep himself very quiet. The effect of the bleeding, we are told, was very decided; for the urine was found on the following day to be transparent and yellow as in health, with acid properties and of a urinous odour: its specific gravity, however, was very low, being only 1.0121. The report of the case closes thus: "complete disappearance of the symptoms. A few weeks afterwards, the urine became again sanguinolent and albumino-fatty, in consequence of fatigue; but two or three days' rest served to restore it to a healthy condition."

---

### RECENT WORKS ON THE PLAGUE.

During the last two or three years there have been published in Paris several works on this frightful pestilence, as it has been observed at different times in Egypt, Syria, Constantinople, and Greece. The names of the chief authors are *Bulard, Clot-Bey, Aubert* and *Gosse.*

There is considerable discrepancy of opinion among these gentlemen on the question how far the prevalence of the disease is, or is not, attributable to the influence of contagion. M. *Aubert* goes so far as to deny the contagiousness of the disease altogether, and endeavours to trace the outbreak and diffusion of it to local causes—the most prominent of which, he says, are the poverty, destitution, and filth of the inhabitants. He thinks that the circumstance—the truth of which we believe is admitted by all observers—of the plague being never entirely extinct during any season in those places where every now and then it rages with pestilential severity, affords a strong argument in favour of his view of the case. But does not the same thing hold true of small-pox? and yet no one denies that *it* is propagated by contagion. We are however, quite willing to admit that, in the case of both these fevers, as well as of other wide-spreading diseases, their diffusion is attributable less to actual propagation from one person to another than to certain atmospheric conditions, the nature of which, however, we are utterly unacquainted with. Nevertheless it seems not improbable that the morbific element, generated in and proceeding from the human body, may act as "the leaven that leaveneth the whole lump," or as the one drop of variolous matter inserted in the arm causes the fermentation, so to speak, of all the fluids of the body, and the consequent production of innumerable pustules on the surface, containing a fluid altogether similar to that used in the primary inoculation.

The truth, therefore, as to the question of the contagiousness or not of the plague seems to lie in the middle between the two extreme opinions. That there is a certain *something* exhaled from the body of a plague, as of a small-pox, patient, and that this exhalation is capable, under favourable circumstances, of producing similar effects in the bodies of other persons exposed to its influence, appears to us to be indisputable; but then it seems equally true that this aerial poison may be widely and generally diffused in a locality at certain times, and may act contemporaneously on all the inhabitants of that locality, only with varying degrees of severity according to the predisposition of different individuals to be affected by it.

The practical deduction to be drawn from this view of the question is this important one—that, while precautionary measures ought certainly to be adopted to prevent the intercourse of the sound with infected persons, there is much in the existing quarantine regulations of most countries that is quite unnecessary, or even positively injurious. If legislators, as well as medical men, were to bear in mind that the laws, which regulate the diffusion of the plague, are essentially the same in nature as those which influence the spreading of small-pox—a pestilence, by the bye, quite as frightful as the plague, even in the worst seasons—fewer errors would have been committed in practice as well as in theory.

Both diseases are communicable from one person to another by direct contact as well as by aerial infection; both are attended with the eruption of cutaneous abscesses, the contents of which are virulently poisonous; both exist in sporadic cases at all times, but are subject to occasional outbreaks of unusual malignancy; and in both, second attacks are of only exceptional occurrence. Would that we might add that the plague, like the small-pox, was capable of being controlled and mitigated by the substitution of another disease! We are not sufficiently acquainted with a complete history of the Oriental pestilence to permit us even to entertain a grounded hope that Providence may raise up a second *Jenner* to arrest its frightful ravages. We do not remember at the present moment if any author has ever ascertained whether the lower animals are affected with an epizotic disease during the prevalence of the plague. It is more than probable that such is the case; but for want of accurate information we cannot say more. It certainly seems to us not at all more unlikely that an antidote may yet be discovered against the plague, than that vaccination has been found to exert such extraordinary preservative and counteracting powers against the spread of small-pox. This hint may not be deemed unworthy of notice, and if it leads to no other result than merely to that of inducing some physician resident in Turkey or Egypt to ascertain whether the brute animals are in any way affected during the prevalence of the plague, the information is not to be despised.

M. *Clot-Bey*, in his description of the disease as he has observed it for several years in Egypt, mentions a fact which very satisfactorily proves that there must be a vitiated state of the atmosphere, acting hurtfully upon all persons; for, he says, that even those, who escape seizure, experience more or less decidedly the precursory symptoms of the disease. "In this condition they complain of glandular pains in the groins and arm-pits, increased by pressure or by muscular exertion, and these are accompanied with loss of appetite, nausea, and feeling of great debility. The expression of the physiognomy is always changed. Those who were thus affected, but still went about their affairs, were in a sort of incessant struggle against the disease, so that it often seemed doubtful whether it or the powers of the system should have the mastery."

Every medical man must have noticed a similar condition during some of the late epidemics of influenza: the disease may not have developed itself fairly in certain persons, but their systems were decidedly suffering from the baneful influence of the atmospheric condition.

The peculiarly characteristic symptom of the plague is the affection of the external glandular system in the groins and arm-pits, occasionally also in the neck and hams, and the formation of carbuncles and petechial spots on the limbs and trunk.

In the mild form of the disease, it usually experiences a favourable crisis by profuse perspiration, accompanied with the resolution or suppuration of the affected glands, within a few days after the attack. On the other hand, in the worst form of the disease, the vital powers seem prostrated from the very first, and death supervenes in a day or two, before any local symptoms are manifested.

But should this not be the case, the primary stage of debility is succeeded by one of re-action, attended with the usual symptoms of typhus fever: the pulse is full, vibratory, and frequent, the face is flushed, the pupils are dilated, the tongue is dry and often horny—in the early stage of the disease it is always white, moist, and of a mother-of-pearl hue—the teeth and lips become black, and the nostrils are full of a pulverulent dark-coloured matter. Jerking of the tendons, hiccup, and coma usually precede death.

In some cases, while the symptoms now described are present, large gangrenous carbuncles make their appearance usually in the neighbourhood of the affected glands, the buboes advance very rapidly to suppuration, the pulse loses its frequency, and a profuse perspiration, and an eruption of erysipelatous patches or of circumscribed abscesses, or hæmorrhage from some passage, form a sort of critical phenomena in the disease.

Dr. *Gosse* describes two forms of plague as he observed it in different parts of Greece. In the *first* the disease seemed to be produced by direct contact, either with the bodies of infected persons or with articles of clothing or bedding which they had used, and then the primary symptoms were local, being not unlike to those of malignant pustule. A carbuncle was gradually developed, and about the second or fourth day a re-action was established around the gangrenous spot: this by degrees became detached without further trouble, save the local symptoms and perhaps also a certain degree of symptomatic fever. When the local re-action did not take place, several buboes appeared in the direction of the lymphatic vessels, all the symptoms became more threatening, and did not abate until the buboes began to suppurate. In the *second* form, the morbific principle, being in a state of greater virulence, had infected the atmosphere to a greater or less extent, and the earliest symptoms were those of constitutional disturbance, indicated by shiverings, vertigo, headache; the development of buboes was a secondary phenomenon, and when glandular swellings advanced to suppuration, the occurrence was observed to be generally of a favourable augury.

The duration of plague varies exceedingly in different cases: it has been known to prove fatal within twelve hours after its invasion, and in some cases it has lasted for twenty or thirty days. Like other pestilential epidemic diseases, the plague seldom exhibits the same features in different seasons. It is usually more malignant in the beginning of an epidemic, becoming less and less severe after it has continued for some time.

The generally-received opinion that those, who once have passed through the plague, are not subject to a second attack seems to be nearly correct; at least as much so in reference to this disease as it is to small-pox. M. *Clot*, however, assures us that he has seen several well-marked cases of second attacks of plague; and Dr. *Gosse* tells us that of forty persons, who had had the disease and were subsequently employed in the lazarettoes, ten suffered from second attacks, of which however only one proved fatal. Does not this circumstance alone argue strongly in favour of the contagiousness of the plague, while at the same time it affords another point of resemblance between it and small-pox?

The pathological anatomy of the plague, if we may use such an expression, is, as might indeed be expected, far from satisfactory. The appearances found after death do not at all explain the formidable symptoms of the disease. It deserves however to be noticed that the examinations made by MM. *Clot* and *Bulard* clearly shew that the whole of the lymphatic system is deeply implicated, and that the internal glands, those for example situated in the mediastina and along the vertebræ and in the pelvis, are all more or less affected, as well as the external glands in the groins and arm-pits. The former of these gentlemen, although a zealous admirer of the Broussaian doctrines, admits that they are certainly not applicable to the explanation of the phenomena of the plague. It is surely too obvious, to require any comments, that the pathology of such a disease as the plague must be sought for after death in the altered state rather of the fluids than of the solids: we should remember that it is the blood that is "touched corruptibly" from the very commencement; and all hopes of attempting a cure must be in enabling the system to eliminate the poison. The channel, in which this is most likely to be effected, appears to be the surface of the body, although the other excretory organs may be acted upon at the same time. It is lamentable to find the very latest writers acknowledging that not the slightest progress has been made in the discovery of any curative plan to control, or even to mitigate, the frightful fatality of the disease. Certainly the most rational method seems to be that of inducing vomiting in the first instance, and then keeping up active determination to the skin by warm saline diaphoretics. The treatment of such diseases as plague, pestilential cholera, and malignant typhus, is to be conducted on nearly the same principles—they are all the product of active poisons introduced into the system, and one of their most prominent symptoms is a vitiated state of the blood. The exhibition of a strong emetic of salt and water, followed by copious draughts of warm water, has often a very marked influence on the future development of such diseases; the circulation, which had been previously weak and oppressed, becomes in consequence fuller and more regular; the skin is brought into a perspiring state; the offending contents of the stomach and duodenum are dislodged and prevented from passing along the intestinal canal, and a downward action of the bowels is frequently induced. The kidneys too are generally stimulated at the same time; we need scarcely add that all the respiratory passages get rid of the mucus that may be obstructing their canals. For these reasons we most strongly recommend that vomiting should be induced in almost every case without exception at the commencement of the attack, and that, if it has come on spontaneously, it should be promoted for some time by the use of copious draughts of tepid salt and water. The attendant will rarely fulfil the full intentions of the physician in this respect; he supposes that if the patient vomits, it is all that is required; far from it; the powerfully-straining efforts, which accompany this act, should be kept up for some time, otherwise their most salutary effects will not be obtained. Every one, who has watched much at the bed-side of patients, knows that in certain cases the matters that are rejected by the first or second acts of vomiting are merely the contents of the stomach, and that no appearance of biliary matter is observed until the straining has been continued for some time. Now in diseases, such as those to which we are alluding, it is most important that, if possible, the gall-bladder and the hepatic and cystic ducts should be well emulged, to use an old and expressive phrase; and of this the only proof is the rejection of their contents upwards. One of the earliest effects of pestilential diseases is unquestionably a vitiated state of the bile—does this arise or not from the greater disturbance of the circulation through the liver in consequence of the immediate proximity of the hepatic veins to the heart? However this may be, it has been remarked by all observers that a copious rejection of bile in the early stages of malignant fever may generally be regarded as a favourable symptom. It will be found too that the action of medicines, and more especially of mercurial preparations, can always be more depended upon in such cases after the free action of vomiting.

Let us suppose now that this has been fully obtained; what is the next thing to be done in such a disease as the plague? We should say, administer effervescing draughts containing the muriate of soda or the chlorate of potash, with or without ammonia according to circumstances, every half hour or hour, until the circulation is equalized and free perspiration is induced. In such a formula we combine the effects of an antiseptic, an aperient, a diuretic, a diaphoretic, and a stimulant; and we need not say how easy it is to vary the proportions of the ingredients so as to make one action predominate over the others, according as the circumstances of the case may require. Between each effervescing draught from five to ten or even twenty grains of calomel with or without camphor should be given, until a drachm or upwards has been administered.

Such is the treatment which, in our opinion, promises the most advantage in pestilential diseases like the plague. As a matter of course, other remedies may be required; such as moderate blood-letting, if the feverish re-action is strong; purgatives, if the bowels are torpid; blisters or sinapisms, when any local distress is severe; stimulants, such as wine and brandy, if the debility is extreme, &c. &c.

As might be expected, the patient is always left exceedingly weak, and his system does not recover its tone of health for several months after a recovery; the use of bark and other tonics, and a change of climate, are generally necessary for the restoration of the strength.—*Rev.*

---

### PREDISPOSING CAUSES OF PULMONARY CONSUMPTION.

Common opinion attributes to the comparative activity of the organs of the body, at different periods of life, their greater or less tendency to become the seat of tuberculous deposition. Thus, in infancy and early youth, when the activity of the encephalon is certainly greater than at any other age, tubercles in the brain or its membranes are not of unfrequent occurrence. Then comes the turn of the abdominal organs, which in youth are endowed with great activity; and every one knows how frequent is disease of the mesenteric glands at this epoch. After puberty, the respiratory organs become more and more developed; and they are certainly by far the most frequent seat of tubercles. This tendency to pulmonary disease continues until middle or advanced life, when "the tuberculous secretion" is usually directed upon the uterus, the mammæ, the prostate, the stomach, &c.

Dr. *Cheneau* has recently published in Paris, a work entitled—*A few words upon this question: can we determine the cause of the predilection of the tuberculous affection for the lungs from the period of puberty to about forty years of age? and can we account for the progressive frequency of phthisis in the present century?*

The following extract from this work, will shew in what manner he endeavours to solve the first of these questions.

"In the early years of life, and up to the age of puberty, Nature seeks only the preservation and the increase of the being. The physical life is concentrated in the organs of reparation, and is altogether assimilatory; and the functional derangements of these organs must therefore favour the circumstances which influence the tuberculous development. Hence the frequency of tubercles in the mesenteric glands and in the bowels during this period of life. A new state of things is observed after puberty. Life, hitherto chiefly physical, becomes then more and more intellectual: sensations, hitherto not felt, influence the whole system; the brain manifests all its powers and tendencies, and in its turn begins to exert a despotic sway over the organs, on which it previously seemed to depend. Besides the direct re-action which the organs of generation exercise on the encephalon, love, with all 'its passions, thoughts, desires,'—not forgetting the too frequent abuse of its delights—fails not to act most deeply on the mind. Then ambition, alas! too frequently disappointed, with hatred, and a host of other racking emotions, add fresh troubles to the troubles of the brain."

This excitement of the brain re-acts, in the opinion of our author, on the lungs through the medium of the eighth pair of nerves, giving rise to the tendency to tuberculous deposition in their structure.—*Gazette Medicale.*

*Remark.*—The very circumstance of genuine phthisis being infinitely more frequent from the 15th to the 40th or 45th year of age than at any other period of life, very naturally suggests the idea that the development and action of the generative organs have something to do with the production of the disease. The manner, in which Dr. *Cheneau* endeavours to explain the connexion, seems to us to be rather circuitous—the re-action of the excited brain through the medium of the eighth pair of nerves inducing an over active state of the organs of respiration. And yet there is the element of truth in this view of the question. For it should be ever kept in mind that not a single strong emotion can be experienced for even a moment, but it is inevitably accompanied with a disturbance, more or less considerable, of the breathing at the time. Witness the laughter of joy, the sobbing of grief, the sighing of chagrin, the panting of hope—what are these phenomena, but so many results of irregular inspiration and expiration? Physiologists have certainly not attended sufficiently to the intimate connexion that exists between mental emotions and their physical effects on the breathing and on the circulation, and thence on every part of the body: the only writer that we remember to have dwelt upon this subject is Mr. *Wardrop*, in his ingenious work on the Heart. He has not applied the reasoning, it is true, to explain the great tendency to pulmonary disease for several years after puberty; but he has pointed out in a very beautiful manner how intense, or long protracted, or frequently recurring, mental emotions must tend to induce cardiac disorders.

When we think of the almost unceasing play of a multitude of feelings and passions that agitate the mind of both sexes between the 16th and the 26th years of age, we may cease to wonder at the frequency of pulmonic derangements—seeing, as we have already said, that every act of mental emotion is almost necessarily accompanied with more or less disturbance in the regularity of the breathing.

There are, no doubt, other agencies to be taken into account, in attempting to explain the marked tendency to tubercular disease fixing itself in the lungs for ten or fifteen years after puberty. The irregular licentious life, too often, of the one sex, and the absurd mismanagement of the other, in reference to their food, clothing, exercise, &c. will not be overlooked by the attentive observer. Of this one thing we may be quite assured, that just in proportion as the artificial mode of existence is pursued, and the further the obvious suggestions of Nature and common sense in the physical education of girls are departed from, so will the tendency to pulmonary consumption be increased. Flannel clothing, high dresses, warm stockings, nourishing diet, taken at proper times of the day and at moderate intervals, early rising, regular exercise in the open air, bathing or sponging the body and brisk friction afterwards, avoiding the heated ball-room, the late hours, and the sentimental excitements of fashionable life—these, simple measures, coupled with a few other precepts which will readily suggest themselves, if more generally adopted, might save the lives of many a tender creature who is annually sacrificed at the shrine of foolish fashion.—(*Rev.*)

---

### New Theory of Tinea; its supposed Vegetable Origin.

A few years ago a young Hungarian physician, M. *Gruby*, announced, as the result of an extensive series of microscopical observations, that the cutaneous disease, known by the name of tinea or favus, is owing to the development and growth in the skin of cryptogamous plants of the genus fungus, which he has called *micoderme.* He tells us that they exhibit the aspect and form of such vegetable productions from their earliest appearance to their complete maturity, at which stage, if divided and examined with a magnifying-glass of two or three hundred powers, he has distinctly recognized the fruits or sporules in the interior of their cavities, as we observe to be the case in other fungi. He is of opinion that the disease is propagated by these seeds. This novel idea of the etiology of the disease has excited much attention among the French dermatologists; and it is but just to M. *Gruby* to acknowledge that the researches, which have hitherto been made on the subject at the St. Louis Hospital in Paris, have tended to confirm the accuracy of his statements.

The treatment, naturally suggested by this theory of the disease, is that of applying caustics or whatever is calculated to destroy the vegetable substance.

M. *Devergie* has, during the last year, been giving an extensive trial to a solution of the acid nitrate of mercury (of the French codex) : the spots become at first of a reddish yellow colour, and subsequently black after the application.

M. *Emery*, of the same hospital, is in the habit of employing a strong solution of iodine for the same purpose, and it has appeared to answer extremely well.

In another article in the same journal, Dr. *Petel* recommends an epilatory ointment; as, in his own opinion, the only prospect of effecting a rapid cure depends upon extracting the hairs from the diseased spots. This gentleman's experience, however, cannot well be trusted to; as he tells us that tinea is never present where the hairs have fallen out, and that the method which nature follows is always by the removal of the hair. He tells us, too, that all internal and external remedies are of little or no use until the hairs are extracted, or fall out spontaneously. It would seem, from what he says, that the empirics, MM. *Mahon*, have more success in the cure of the disease than any of the regular practitioners in Paris : their plan consists in applying, first, a strong lixirial soap to the head, and then an epilatory plaster, which, when removed, draws the hairs off with it.

Dr. *Pelet* recommends the following formula for an epilatory ointment and powder:—

| | | |
|---|---|---|
| Take of | Soda (of commerce) .. | 60 parts. |
| | Slaked lime .. .. .. .. | 4 „ |
| | Lard .. .. .. .. .. .. | 120 „ |
| | Mix together. | |

| | | |
|---|---|---|
| Take of | Quicklime.. .. .. .. .. | 120 parts. |
| | Powdered charcoal .. .. | 8 „ |
| | Mix together. | |

After cutting the hair very short, the crusts are to be detached by applying linseed poultices to the scalp, and washing it repeatedly with soap and water. After several days' use of these means, all the affected parts are then to be rubbed with the ointment: this is to be repeated daily. The swelling and redness of the scalp gradually subside, but without ever ceasing entirely. The pustules, the successive reproduction of which keeps up the disease, become more rare and appear only at long intervals. To effect this change, from two to three or four months are often required. When this is the case, a pinch of the powder is to be sprinkled among the hair every second day or so. The hair becomes gradually loosened, so that it can be easily detached with the fingers or with a forceps, without causing pain. When the affected parts are rendered quite bald, the treatment is nearly at an end; all that is necessary is to anoint the head with a small portion of the ointment every two or three days, and to keep it exceedingly clean.—*Bulletin de Therapeutique.*

*Remarks.*—It has often occurred to us that tinea, at least some forms of it, might be dependent upon the presence of some animalculæ in the affected parts: the local nature of the eruption, and its entire independence of constitutional derangement, coupled with the inveterate obstinacy of the disease, have probably suggested this idea to our mind.

Whether M. *Grube's* announcement of its vegetable origin be correct, remains to be proved; the question, we hope, will be attentively examined by some good microscopic observer. The notion, that not a few diseases may be owing to the development of animal or vegetable germs in the body, appears to us to be not at all improbable. The subject of the generation of entozoa is certainly a very curious one; but as yet we are at the very threshold of the enquiry.

